# NIR-II-Activated iridium single-atom nanozymes for synergistic antibacterial therapy and tissue regeneration in MRSA-infected wounds and acute lung injury

**DOI:** 10.1016/j.bioactmat.2025.05.022

**Published:** 2025-05-24

**Authors:** Danyan Wang, Hui Jin, Yetao Shen, Dandan Wang, Jingjing He, Jinmiao Qu, Xiaojun He, Zhengli Jiang

**Affiliations:** a Department of Pharmacy, Taizhou Hospital of Zhejiang Province Affiliated to Wenzhou Medical University, Taizhou, Zhejiang, 317000, China; b Department of Ophthalmology, Peking University First Hospital, Beijing, 100034, China; c Taizhou Key Laboratory of Pharmaceuticals Therapy and Translation Research, Taizhou, Zhejiang, 317000, China; d State Key Laboratory of Ophthalmology, Optometry and Visual Science, Eye Hospital, Wenzhou Medical University, Wenzhou, Zhejiang, 325027, China; e Department of Thyroid Surgery, National Key Clinical Specialty (General Surgery), The First Affiliated Hospital of Wenzhou Medical University, Wenzhou, Zhejiang, 325000, China

**Keywords:** Methicillin-resistant *Staphylococcus aureus*, Photothermal, Peroxidase-like activity, Iridium-single-atom, Anti-infection therapy

## Abstract

Methicillin-resistant *Staphylococcus aureus* (MRSA) remains a major pathogen in ventilator-associated pneumonia and wound infections. To address the limitations of traditional antibiotics, we developed a novel iridium-based single-atom catalyst (Ir/CN SAC) anchored on a nitrogen-doped carbon matrix. Engineered for ultra-low metal loading and maximal active site exposure, this catalyst integrates robust photothermal and catalytic functionalities. Under second near-infrared (NIR-II, 1270 nm) irradiation, the Ir/CN SAC efficiently converts light to heat and catalytically generates reactive oxygen species (ROS), achieving a potent photothermal-catalytic synergistic effect. This dual-action mechanism enabled rapid bacterial eradication *in vitro* and significantly accelerated wound healing and lung tissue repair in MRSA-infected *in vivo* models. Transcriptomic analyses revealed downregulation of pro-inflammatory pathways, shedding light on the immunomodulatory roles of the treatment. Notably, the Ir/CN SAC exhibited negligible toxicity and enhanced peroxidase-mimicking activity via thermal activation. Collectively, the Ir/CN SAC presents a promising strategy for treating MRSA infections in wounds and the lungs via a synergistic treatment model.

## Highlights

• We created Ir/CN SAC characterized by maximum active site exposure.
• Ir/NC SAC possesses excellent photothermal properties and peroxidase activity.
• Ir/NC SAC could significantly kill bacteria and eradicate biofilm *in vitro.*
• Ir/NC SAC promoted pneumonia recovery via photothermal synergistic peroxidase.
• Ir/NC SAC exhibits good biocompatibility and biosafety.

## Graphical abstract

**Figure:**
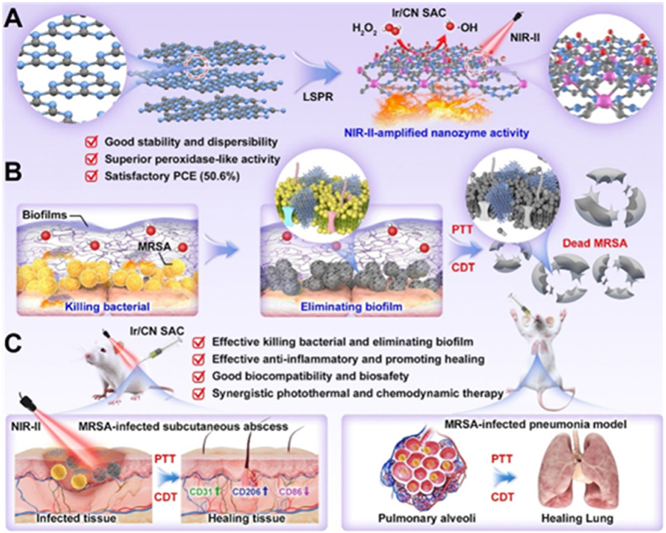

## 1 Introduction

Bacterial pneumonia remains a major global health threat, particularly for vulnerable groups such as young children and elderly individuals with weakened immune systems [1]. This condition is often associated with microbial imbalances in the lungs and immune dysfunctions. MRSA, a strain of Gram-positive bacteria, is a significant contributor to hospital-acquired infections, especially those affecting the respiratory system and ventilator-associated pneumonia [2], while it also contributes largely to skin and soft tissue infections [[3], [4], [5], [6]], osteomyelitis, and bacteremia [[7], [8], [9]]. Although antibiotics are still the primary means of treating MRSA-infected wounds and acute lung injury [10,11], their excessive use contributes to the rapid emergence of resistant strains [[12], [13], [14]]. Developing new antibiotics is a lengthy and expensive process, taking over ten years and billions of dollars [15]. Additionally, antibiotics can inhibit immune cells' ability to eliminate pathogens [16,17] and disrupt the lung microbiome [18]. With the growing threat of antibiotic resistance and the slow pace of new drug discovery, the availability of effective antibiotics is dwindling [19,20]. Therefore, there is an urgent need for alternative therapeutic strategies that target bacterial infections, particularly Gram-positive bacteria, without causing resistance.

Recent advances in antimicrobial agents that rely on ROS-mediated mechanisms have sparked significant interest. ROS can destroy bacteria without causing the resistance seen with traditional antibiotics [21,22]. Nanozymes, which facilitate the production of ROS, have been identified as promising alternatives [[23], [24], [25], [26], [27], [28]]. These ROS include reactive molecules like hydroxyl radicals, superoxide anions, singlet oxygen, and hydrogen peroxide [29]. Nanozymes with peroxidase (POD)-like functions can convert H_2_O_2_ into highly reactive hydroxyl radicals, effectively killing bacteria or dismantling biofilms in a process known as chemodynamic therapy (CDT) [26,[30], [31], [32], [33]].

Single-atom catalysts (SACs) are materials with isolated metal atoms on solid substrates, providing numerous exposed active sites that enhance catalytic performance compared to nanoparticles [34,35]. SACs achieve nearly 100 % atom utilization [36,37], representing the peak of atomic-level material design [38]. However, the catalytic performance is largely determined by surface atoms, with minimal contribution from the inner core [37]. Consequently, strategically placing metal atoms on the outer surfaces of supports can optimize catalytic efficiency, reduce metal consumption, and minimize toxicity in biomedical settings [39].

The intrinsic activity of the metal is more crucial in determining the catalytic efficacy of SACs than the method of metal loading. Iridium (Ir) is a highly stable, corrosion-resistant metal known for its excellent catalytic properties [39,40]. Ir (III) complexes have recently gained attention for their potential in chemotherapy [[41], [42], [43], [44]], though the application of Ir-based nanocatalysts in biomedicine is still under investigation [45].

The Arrhenius equation states that increasing the temperature increases the catalytic activity of nanozymes and encourages the production of ROS [46]. POD-like activity in conjunction with photothermal therapy (PTT) is a viable remedy for the escalating issue of antibiotic resistance. Although PTT is a regulated, non-invasive treatment for bacterial infections [47], overheating can harm healthy tissues [[48], [49], [50], [51], [52]]. The NIR-II window laser has a greater skin tolerance threshold and more tissue penetration than the NIR-I window laser [53]. For *in vivo* applications, the NIR-II laser is more suitable. Therefore, PTT and peroxidase activity can be combined to increase ROS generation and reduce tissue damage.

**Scheme 1:**
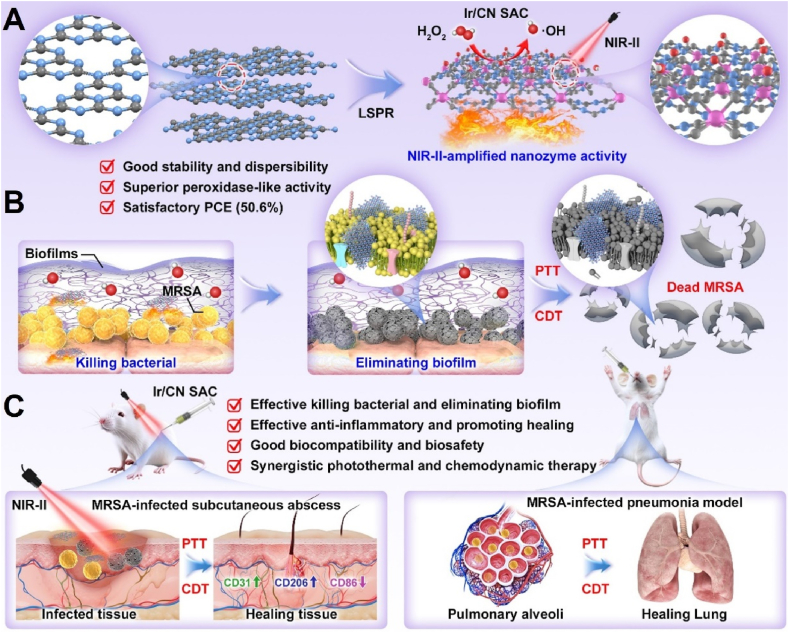
(A) Overview of how Ir/CN SAC is synthesized. (B) Application of the NIR-II-enhanced nanozyme strategy for effective MRSA elimination and biofilm destruction *in vitro*. (C) Successful treatment of deep subcutaneous abscesses and bacterial pneumonia *in vivo* by combining photothermal and chemodynamic therapies.

In this study, we developed an Ir-based single-atom catalyst (Ir/CN SAC) supported on a nitrogen-enriched carbon composite. This material exhibited robust peroxidase activity when exposed to heat, generating high levels of ROS. The Ir/CN SAC demonstrated a high photothermal conversion efficiency (PCE) of 50.6 % at 1270 nm NIR-II light and excellent photothermal stability. It also demonstrated superior POD-like activity with a Michaelis-Menten constant (*K*_m_ = 0.69 mM). The combination of PTT and CDT effectively targeted Pseudomonas aeruginosa (PAO1) and MRSA in laboratory tests and promoted wound healing in MRSA-infected models, as well as aiding recovery from acute lung injuries *in vivo* (see Scheme 1). Toxicological assessments showed negligible toxicity at the doses tested. This research demonstrates the potential of Ir/CN SAC as a versatile and effective therapeutic platform for MRSA infections through a synergistic treatment approach.

## 2 Experimental procedure

### 2.1 Chemicals and materials

Aladdin supplied reagents such as 3, 3, 5, 5-tetramethylbenzidine (TMB) and O-phenylenediamine (OPD). Solarbio provided culture media including agar, Tryptic Soy Broth (TSB), and LB Broth (LB), while Thermo Fisher Scientific contributed phosphate-buffered saline (PBS). A Hitachi U-3100 spectrophotometer was used to measure UV–vis–NIR absorption spectra, and the structural analysis of Ir/CN SAC was conducted using a Talos F200S transmission electron microscope (TEM) from Thermo Fisher Scientific. The oxidation states of elements were examined with Thermo Scientific K-Alpha X-ray photoelectron spectroscopy (XPS). Using 100 μL of Ir/CN SAC aqueous suspension (100 μg/mL, PH = 5.5), inductively coupled plasma-mass spectrometry (ICP-MS) (Agilent) was used to measure the release of iridium ions both before and after light exposure. A Nikon Japan A1 confocal microscope (CLSM) was used for fluorescence imaging.

### 2.2 Synthesis of Ir/CN SAC

Briefly, 50 mL of methanol was used to dissolve 1.785 g of Zn(NO_3_)_2_•6H_2_O (6 mmol). The Zn(NO_3_)_2_•6H_2_O solution was then vigorously stirred for 5 min at room temperature before receiving an additional 50 mL of methanol solution containing 1.970 g of 2-methylimidazole (24 mmol). After that, the combination solution was kept for 2 h at room temperature. The precipitates were then centrifuged, cleaned, and dried at 80 °C. After dispersing the produced particles in 100 mL of methanol solution, 100 mg of the Ir(acac)_3_ solution were made and added dropwise to the aforementioned powder solution. The resultant solution was recovered by centrifugation, cleaned six times with methanol, and vacuum-dried overnight after being violently stirred for 2 h to carry out the ionic exchange procedure. After that, the powder was heated to 900 °C for 3 h in a tube furnace with argon gas flowing at a rate of 5 °C min^−1^ (100 mL min^−1^). Ir/CN SAC was obtained by cooling the resultant powder to room temperature.

### 2.3 Photothermal performance and photostability of Ir/CN SAC

A FLUKE thermal camera was employed to initially evaluate Ir/CN SAC, capturing temperature variations in real time over a designated period. To investigate the material's photothermal properties, a 1270 nm laser (1 W/cm^2^, 6 min) was directed at 100 μL of Ir/CN SAC solution with varying concentrations. Subsequently, a 1270 nm laser was used to irradiate a 100 μL suspension of Ir/CN SAC (100 μg/mL) at different power levels while continuously monitoring temperature fluctuations.

The photostability of Ir/CN SAC was examined using an identical approach over five cycles. A 1270 nm laser (1.0 W/cm^2^) was applied to a 100 μL aqueous suspension of Ir/CN SAC (100 μg/mL) for 6 min, followed by natural cooling after the laser was switched off. Finally, a 100 μL aqueous suspension of Ir/CN SAC (100 μg/mL) underwent periodic exposure to a 1270 nm laser (1.0 W/cm^2^) until a stable temperature was achieved. The laser was then deactivated to allow natural cooling, facilitating the evaluation of Ir/CN SAC's photothermal conversion efficiency. During the cooling phase, dynamic temperature readings were taken every 20 s. The photothermal conversion efficiency (η) was calculated following the methodology outlined in prior research [54].

### 2.4 POD-like property of Ir/CN SAC

•OH indicators were 1.0 mM TMB and OPD dissolved separately in dimethyl sulfoxide (DMSO). Different concentrations of Ir/CN SAC solutions (100 μg/mL, pH = 5.5) were mixed with H_2_O_2_ (1.0 mM) and TMB for 3 min, followed by absorbance measurement using UV–vis–NIR spectroscopy. The absorbance was tracked at 652 nm for the oxTMB product and at 420 nm for oxOPD, after incubation at 25 °C for different time points (0–6 min). The mixture of Ir/CN SAC, H_2_O_2_, and OPD was exposed to radiation for 0–8 min to evaluate photothermally enhanced POD-like activity. Absorbance at 420 nm was recorded via UV–vis–NIR spectroscopy. Ir/CN SAC's pH-dependent catalytic activity was assessed using citric acid and disodium hydrogen phosphate.

### 2.5 Chemical kinetics analyses

Ir/CN SAC (100 μg/mL) chemical kinetics were examined using TMB (1 mM) or OPD (1 mM). Absorbance at 652 nm or 420 nm was recorded after adding varying amounts of H_2_O_2_ (1, 2, 4, 8, 12, 16 mM). Since Ir/CN SAC is a dual substrate, different concentrations of TMB or OPD (1, 2, 4, 8, 12, 16 mM) with H_2_O_2_ (1 mM) were then utilized for detection. The high maximum reaction velocity (*V*_max_) and *K*_m_ values were determined using the Lineweaver-Burk plot, derived from the Michaelis-Menten curve [55].

### 2.6 Antibacterial effect of Ir/CN SAC *In vitro*

The antibacterial activity of Ir/CN SAC was tested with PAO1 and MRSA bacteria. The bacterial solution was split into eight groups: PBS, PBS + NIR-II, H_2_O_2_, H_2_O_2_ + NIR-II, Ir/CN SAC, Ir/CN SAC + NIR-II, Ir/CN SAC + H_2_O_2_, and Ir/CN SAC + H_2_O_2_ + NIR-II, with three measurements per group. The concentrations of Ir/CN SAC, H_2_O_2_, and bacteria were 100 μg/mL, 100 μM, and 2 × 10^7^ CFU/mL. After 5 min of exposure to 1270 nm NIR-II laser light (1.0 W/cm^2^), Groups II, IV, VI, and VIII were incubated for 2 h at 37 °C.

For viability assessment, the number of surviving colonies was counted after plating 20 μL of the diluted bacterial solution (10^3^ times) onto agar and incubating for 15 h at 37 °C. Simultaneously, different subgroups of bacterial cultures were co-stained with a green fluorescent dye (SYTO-9) and a red fluorescent dye (PI) for 20 min, and a confocal microscope (Nikon) was used to evaluate the survival of bacteria again. Scanning electron microscopy (SEM) was used to inspect the bacterial morphology and Ir/CN SAC presence. The bacterial solution was fixed with 4 % paraformaldehyde for 4 h, followed by gradient ethanol dehydration and analysis of dried bacteria on gold-coated conductive tape. A bicinchoninic acid (BCA) protein assay kit was used to perform MRSA protein leakage assays after various treatments to further evaluate the loss of membrane integrity. The SpectraMax Plus 384 microplate reader was used to measure the absorbance at 562 nm. Next, using the standard curve, the precise amount of MRSA protein leakage was determined.

### 2.7 Bacterial biofilm assay

MRSA or PAO1 (20 μL, 1 × 10^7^ CFU/mL) was added to a 24-well plate to create the biofilm, which was then allowed to develop at 37 °C for the entire night. After 24 h, the floating bacterial biofilms were removed by carefully discarding the top layer (TSB medium) of the bacterial solution. In the same way, biofilms were formed in eight groups: PBS, PBS + NIR-II, H_2_O_2_, H_2_O_2_ + NIR-II, Ir/CN SAC, Ir/CN SAC + NIR-II, Ir/CN SAC + H_2_O_2_, and Ir/CN SAC + H_2_O_2_ + NIR-II. To fix the biofilms, the supernatant from every well in every group was taken out and dried. After fixation, the biofilms were stained with 200 μL of 5 % crystal violet dye. After 30 min of staining, biofilms were rinsed twice with PBS to get rid of any remaining crystal violet. To assess the survival rate of bacterial biofilm, 200 μL of 33.3 % acetic acid was used to dissolve the dye retained in bacterial biofilms. Absorbance was then measured at 590 nm using a microplate reader. Additionally, a live/dead dual fluorescence staining technique (calcein-AM/PI) was applied, and after 15 min of staining, biofilms were visualized under a Nikon Eclipse Ti A1 confocal fluorescence microscope.

### 2.8 Cytotoxicity of Ir/CN SAC *In vitro*

Cytocompatibility of Ir/CN SAC was assessed using Cell Counting Kit-8 (CCK-8) and mouse epithelioid fibroblasts (L929 cells). Cell viability was then measured according to previous literature [28].

### 2.9 *In vivo* antibacterial activity using MRSA abscess model

All animal experiments were conducted in accordance with Wenzhou Medical University animal care protocols and IACUC/AAALAC standards. MRSA infections were induced via subcutaneous injection in female Balb/c mice (5–6 weeks old). After 24 h, the infected mice were randomly assigned into four treatment groups (n = 5 per group): (I) PBS + NIR-II, (II) Ir/CN SAC + NIR-II, (III) Ir/CN SAC + H_2_O_2_, and (IV) Ir/CN SAC + H_2_O_2_ + NIR-II. Infected regions were subcutaneously injected with 100 μg/mL of Ir/CN SAC solution, and groups (III) and (IV) were simultaneously added with H_2_O_2_ (0.1 mM). NIR-II laser irradiation (1270 nm, 1 W/cm^2^, 5 min) was applied in groups (I), (II), and (IV), with temperature variations monitored using a FLUKE thermal imager. Imaging was performed on days 0, 1, 3, 5, 7, and 10 post-treatment. On day 10, skin tissues were collected for histological analysis, including immunofluorescence, Masson staining, hematoxylin-eosin (H&E) staining, and Gram staining. Major organs were also harvested and examined via H&E staining.

### 2.10 Antibacterial activity MRSA-infection pneumonia model *In vivo*

Following anesthesia, each mouse received 50 μL of 2 × 10^7^ CFU/mL MRSA via intratracheal instillation mediated by intubation. The mouse was kept upright for 2 min after the bacterium injection to give the bacteria time to enter the lung. Five mice per group were randomly assigned to one of four groups: (I) PBS + NIR-II, (II) Ir/CN SAC + NIR-II, (III) Ir/CN SAC + H_2_O_2_, and (IV) Ir/CN SAC + H_2_O_2_ + NIR-II. After 4 h. Isoflurane (1.5 %–2.5 %) was used to anesthetize the infected mice as part of the treatment. The mice were then given either Ir/CN SAC (100 μg/mL) or PBS (200 μL) via the respiratory system, and groups (III) and (IV) were simultaneously added with H_2_O_2_ (0.1 mM). For 5 min, the mice in groups I and IV had their chests lit by an NIR-II laser (1270 nm, 1 W/cm^2^).

Using the enzyme-linked immunosorbent assay (ELISA), histology assay, and the traditional plate count method, the mice's lungs were removed and studied when they were killed 24 h later.

### 2.11 TGF-β, IL-10, IL-6, and TNF-α measurement

Initially, bronchoalveolar lavage fluid (BALF) was made using the previously described procedure. Each group's mice had 1.5 mL of cool, sterile PBS instilled into their lungs. After centrifuging the collected BALF for 10 min at 2400 rpm, the precipitate and supernatant were separated. Sterile PBS was used to redisperse the precipitate in BALF. Thermo Fisher ELISA kits were used to measure the levels of transforming growth factor-β (TGF-β), interleukin-10 (IL-10), interleukin-6 (IL-6), and tumor necrosis factor-alpha (TNF-α) in the BALF supernatant.

### 2.12 Hematological assessment

Three healthy mice received intraperitoneal injections of PBS or Ir/CN SAC (100 μL, 100 μg/mL). After 24 h, they were euthanized, and blood was collected from the ocular veins for analysis.

### 2.13 Hemolytic potential of Ir/CN SAC

The haemolytic effect of Ir/CN SAC was determined by centrifugation of the lower erythrocytes using fresh mouse blood. After three PBS washes, the resulting erythrocyte suspension was diluted in PBS, and then 500 μL of the erythrocyte suspension was mixed with 500 μL of Ir/CN SAC suspension at different concentrations, and the mixture was incubated for 4 h at 37 °C. After the reaction was completed, the mixture was centrifuged, and the absorbance of the supernatant was measured at 540 nm. The percentage haemolysis was calculated according to the formula previously described [56].

### 2.14 Statistical evaluation

Every experiment was carried out independently at least three times, and the mean ± standard deviation was used to express the results. Using Student's t-tests in GraphPad Prism 10, statistical significance was established for comparisons between several groups. The following were the statistical significance levels: ∗*p* < 0.05, ∗∗*p* < 0.01, and ∗∗∗*p* < 0.001.

## 3 Results and discussion

### 3.1 Synthesis and structural Characterization of Ir/CN SAC

**Fig. 1:**
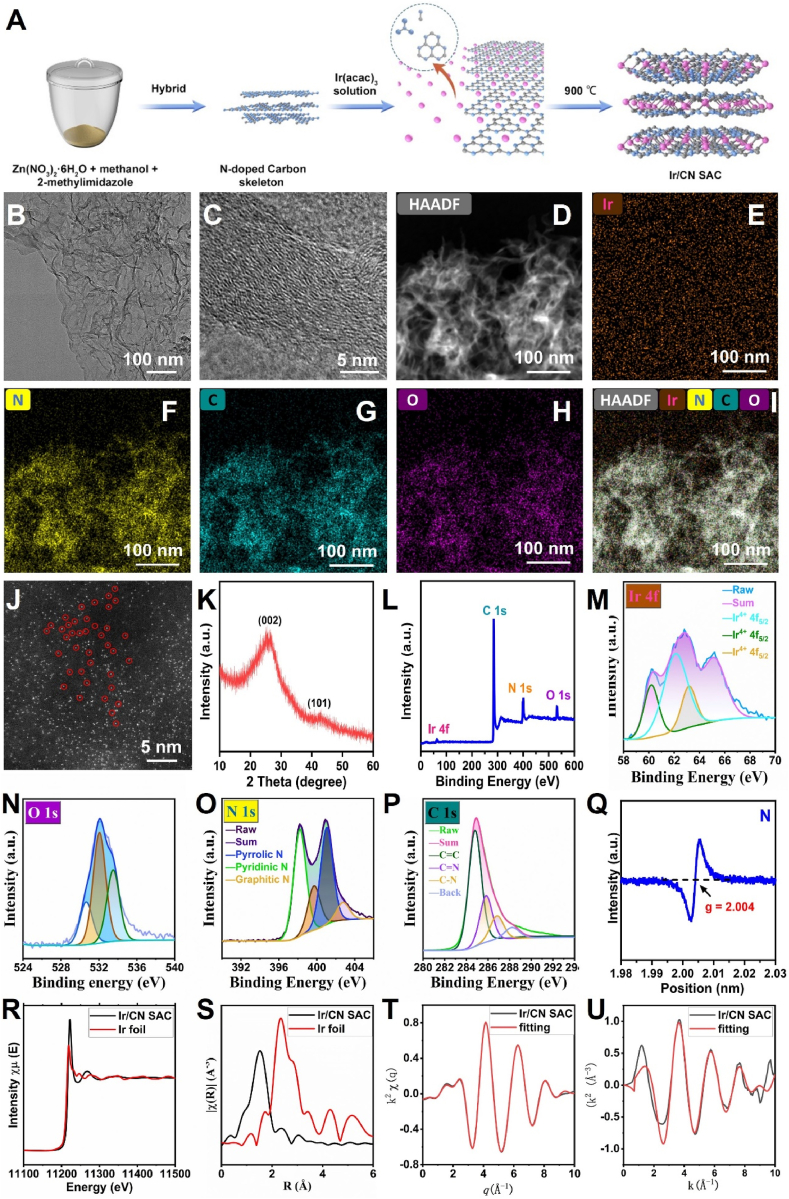
Characterization of Ir/CN SAC structure and composition. (A) A flowchart of Ir/CN SAC fabrication. (B) TEM image, (C) TEM representation of synthesized Ir/CN SAC with a 5 nm scale bar. (D–I) Elemental mapping of Ir, N, C, and O distribution in Ir/CN SAC using HAADF-STEM-EDS. (J) High-resolution HAADF-STEM image showing isolated Ir atoms. (K) XRD pattern (JCPDS NO.06-0598). (L) Full XPS spectrum of Ir/CN SAC. (M) XPS of Ir 4f region, (N) O1s region, (O) N 1s region, and (P) C 1s region. (Q) EPR spectra. (R) Ir K-edge XANES and (S) EXAFS spectra of Ir/CN SAC compared to Ir foil. (T) EXAFS fitting results in q-space. (U) EXAFS fitting results in k-space.

A single-atom catalyst with maximized active site exposure was designed to enhance catalytic activity. As illustrated in Fig. 1A, Ir/CN SAC synthesis involved three critical steps: in situ assembly of metal-organic units, ionic exchange deposition of Ir atoms, and high-temperature pyrolysis to obtain the final product [57]. The nitrogen-doped carbon framework facilitated Ir atom loading during the ion-exchange process. TEM analysis confirmed that Ir/CN SAC exhibited a sheet-like morphology (Fig. 1B), while high-resolution TEM (HRTEM) revealed a smooth nanosheet surface (Fig. 1C).

Elemental distribution was investigated using energy-dispersive X-ray spectroscopy (EDS) mapping and high-angle annular dark-field scanning TEM (HAADF-STEM). Dark-field imaging of individual Ir/CN SAC particles showed uniform contrast (Fig. 1D), while EDS mapping indicated homogeneous dispersion of Ir, N, C, and O without phase separation (Fig. 1E–I).

The aberration-corrected scanning transmission electron microscope (AC-STEM) was then used to confirm the dispersity of Ir atoms. High metal loading and the existence of isolated Ir atoms in the Ir/CN SAC catalyst are indicated by the high density of bright white dots in Fig. 1J. This event demonstrates that single Ir atom active sites arise without iron nanocluster or nanoparticle agglomeration. The two significant peaks with 2θ values of 25° and 43.8° corresponded to the crystal planes (002) and (101) of Ir/CN SAC, as shown by the X-ray diffraction (XRD) results of Ir/CN SAC (Fig. 1K). This may be attributed to the conversion of the organic carbon of the N-doped Carbon skeleton to crystalline CN. The exceptional purity of the final Ir/CN SAC product was demonstrated by the lack of noticeable impurity peaks. Raman spectroscopy was used to further validate the graphitic structure of CN (Fig. S1). Furthermore, Ir, C, N, and O were detected in the Ir/CN SAC XPS survey spectrum (Fig. 1L). The elemental makeup and chemical environment of Ir/CN SAC were examined using XPS. The XPS survey revealed that the weight content of Ir (Fig. 1M), O (Fig. 1N), N, and C was calculated to be 0.23 %, 4.23 %, 15.32 %, and 80.17 %, respectively, in accordance with the findings of the EDS mapping. Numerous active sites for attaching Ir atoms were made available by the high quantity of N. The Ir-N coordination was validated by the binding energy peak at 398.53 eV in the high-resolution N 1s XPS spectra (Fig. 1O). Additionally, it was possible to match four distinct peaks that corresponded to graphitic N, pyridinic N, pyrrolic N, and oxidized N. For the Ir-Nx moieties, the pyrrolic and pyridinic N may serve as attachment sites. The C=C, C=N, and C-N bonds were identified by the three peaks that were found in the C 1s spectra (Fig. 1P). The electron paramagnetic resonance (EPR) spectra of Ir/CN SAC are displayed in Fig. 1Q. It reveals two peaks, which indicate high-density defects. Furthermore, the intensity of the iridium L3-edge (Fig. 1R) can be used to characterize the average oxidation state of Ir4+ species with partial oxidation, as shown by the X-ray absorption near-edge structure (XANES) spectra. XPS observations also validated the calculated oxidation state of iridium in Ir/CN, which is 4.0 based on the XANES spectra. The single-atom dispersion of iridium species in Ir/CN SAC was further validated by excluding Ir-Ir interactions using Fourier-transformed extended X-ray absorption fine structure (EXAFS) analysis (Fig. 1S–U). When combined, these findings verified that a single-atom distributed Ir/CN SAC was successfully synthesized.

### 3.2 Evaluation of Ir/CN SAC photothermal performance

**Fig. 2:**
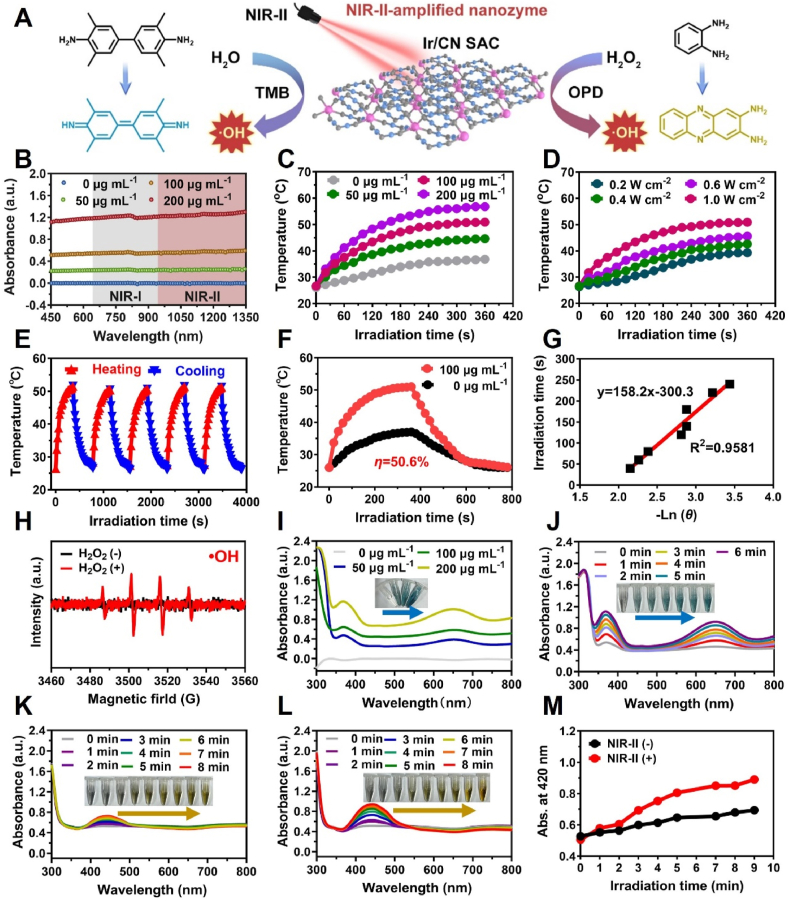
Analysis of Ir/CN SAC's POD-like activity, optical properties, and photothermal behavior. (A) Mechanistic diagram of Ir/CN SAC's peroxidase activity. (B) Absorption spectra of Ir/CN SAC suspension at different concentrations. (C) Temperature profiles of different concentrations of Ir/CN SAC under laser irradiation at 1270 nm (1.0 W/cm^2^). (D) Temperature variations of Ir/CN SAC suspension (100 μg/mL) at varying power densities. (E) Temperature response over five laser on/off cycles. (F) Heating and cooling profiles of Ir/CN SAC at different concentrations. (G) Cooling kinetics of Ir/CN SAC suspension. (H) A typical ESR spectrum of hydroxyl radical generated from the Fenton reaction and trapped by DMPO. Ir/CN SAC's POD-like characteristic with (I) TMB as a substrate. Color variations of related samples are shown in the digital photos inset. Absorption spectra of TMB (J) and OPD (K) in Ir/CN SAC suspension (100 μg/mL) + H_2_O_2_ + TMB/OPD reaction systems as a function of time. OPD absorption spectra in reaction systems including Ir/CN SAC suspension (100 μg/mL) + H_2_O_2_ + OPD + NIR-II, as a function of time (L). (M) Ir/CN SAC's enhanced POD-like characteristic when exposed to NIR-II radiation.

The photothermal properties of Ir/CN SAC were evaluated using UV–vis–NIR absorption spectroscopy, which demonstrated broad absorption across the NIR-I and NIR-II regions, confirming its suitability as a photothermal agent (PTA) (Fig. 2B). When exposed to 1270 nm (1.0 W/cm^2^) NIR-II laser irradiation, suspensions containing varying concentrations of Ir/CN SAC exhibited rapid temperature increases within 6 min, reaching 44.6 °C, 50.9 °C, and 56.8 °C, respectively, whereas pure water temperature remained below 37 °C (Fig. 2C). FLUKE thermal imaging (Fig. S2) captured real-time images of the temperature elevation.

Further investigation demonstrated a power-dependent photothermal response, with temperatures rising to 39.2 °C, 42.6 °C, 45.6 °C, and 50.9 °C as laser power density increased from 0.2 W/cm^2^ to 1.0 W/cm^2^ at a fixed Ir/CN SAC concentration (100 μg/mL) (Fig. 2D), demonstrating a dose-dependent photothermal effect.

Heating and cooling cycle experiments were conducted to assess the photothermal stability of Ir/CN SAC under NIR-II irradiation (1270 nm, 1.0 W/cm^2^) (Fig. 2E). In comparison to the results obtained under 808 nm (Figs. S3A and B) and 1064 nm (Figs. S3C and D), which were 31.9 % and 41.6 %, respectively, the cooling profiles showed a photothermal conversion efficiency (η) of 44.1 % for Ir/CN SAC under 1270 nm (Fig. 2F and G). From the above experimental results, Ir/CN SAC displayed superior photothermal conversion efficiency and deeper tissue penetration, establishing its potential as an effective photothermal agent for PTT.

### 3.3 Increased peroxidase-like activity of Ir/CN SAC by NIR-II laser

To further verify the peroxidase-mimicking capacity of Ir/CN SAC, substrates such as TMB and OPD were utilized for assessment (Fig. 2A). Then, EPR was used to directly detect •OH radicals. As shown in Fig. 2H, the 5,5-dimethyl-1-pyrroline-N-oxide (DMPO) trapped radical signature clearly confirmed the production of a high amount of •OH in a mixture containing Ir/CN SAC (100 μg/mL) and H_2_O_2_ (1 mM). Absorbance measurements at 652 nm demonstrated the transformation of TMB into its oxidized form (oxTMB), which increased progressively with a rise in Ir/CN SAC concentration (0–200 μg/mL) and extended reaction time (0–6 min), as shown in Fig. 2I and J. A parallel effect was observed when OPD was used as the substrate, generating a novel absorption peak at 420 nm (Fig. 2K), highlighting the peroxidase-like properties of Ir/CN SAC.

Temperature and pH also affect the catalytic activity of nanozymes [54]. The buffer's pH range of 4.0–6.0 ensures a high peroxidase-like catalytic performance of Ir/CN SAC, according to various phosphate and sodium citrate ratios (Fig. S4). To assess the influence of NIR-II irradiation on this catalytic activity, OPD absorption was measured in both non-irradiated and irradiated (Fig. 2L) environments. As depicted in Fig. 2M, exposure to NIR-II significantly amplified Ir/CN SAC's peroxidase activity. Consistent with the results of Figs. S5A-S5D, the peroxidase-like activity was significantly enhanced under 1270 nm laser irradiation. Subsequently, the steady-state kinetic behavior was examined using varying H_2_O_2_ concentrations (Figs. S6A and S6B), with the *K*_m_ and *V*_max_ derived from the Lineweaver-Burk plot. The results demonstrated superior catalytic kinetics with Ir/CN SAC, reflected in lower *K*_m_ (0.69 mM) and higher *V*_max_ (4.2 × 10^−7^ M/s) values (Fig. S6C) compared to traditional HRP enzyme data [58] (Table S1), indicating the enhanced interaction of Ir/CN SAC with H_2_O_2_ and its role as an efficient nanozyme. Then the steady-state kinetics of Ir/CN SAC was further tested using different concentrations of TMB or OPD, and Ir/CN SAC showed substrate concentration-dependent characteristics (Figs. S7A-S7D).

### 3.4 *In vitro* antibacterial performance of Ir/CN SAC

To investigate the antibacterial potential of Ir/CN SAC and its synergistic effects with H_2_O_2_, colony counting methods were used to quantify bacterial viability against MRSA and PAO1. Prior to this, the cytotoxicity of varying concentrations of Ir/CN SAC and H_2_O_2_ was evaluated to mitigate potential harm to living organisms. As shown in Fig. S8, the release of iridium ions was quantified by ICP-MS. Minimal antibacterial effects were observed with Ir/CN SAC (100 μg/mL) and H_2_O_2_ (0.1 mM) alone, as shown in Figs. S9A-D.

**Fig. 3:**
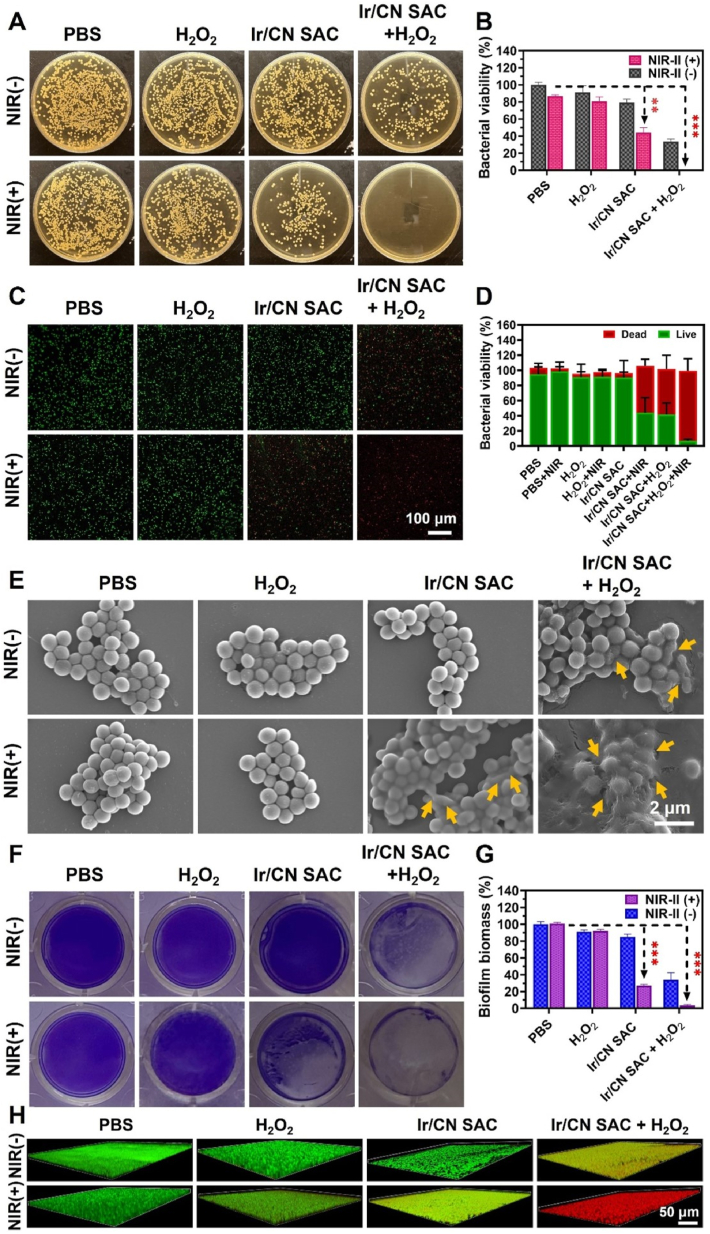
Ir/CN SAC's antibacterial activity *in vitro*. (A) Pictures of a normal agar plate containing colonies of MRSA pathogens after different treatments. (B) Quantitative data pertaining to the MRSA survival rate after treatments, as established by the conventional plate counting technique. The live/dead staining assay and the corresponding quantitative statistics are shown in (C) and (D), where green/red fluorescence indicates live or dead bacteria. (E) SEM pictures of MRSA following various treatments. Changes from the MRSA's initial morphology are indicated by yellow arrows. (F) Pictures of the MRSA biofilm and (G) quantitative data on the biofilm's biomass after treatment. (H) MRSA biofilm confocal pictures after different treatments. Three correlative studies provided the results (Mean ± SD, n = 3, ∗∗*p* < 0.01, ∗∗∗*p* < 0.001).

**Fig. 4:**
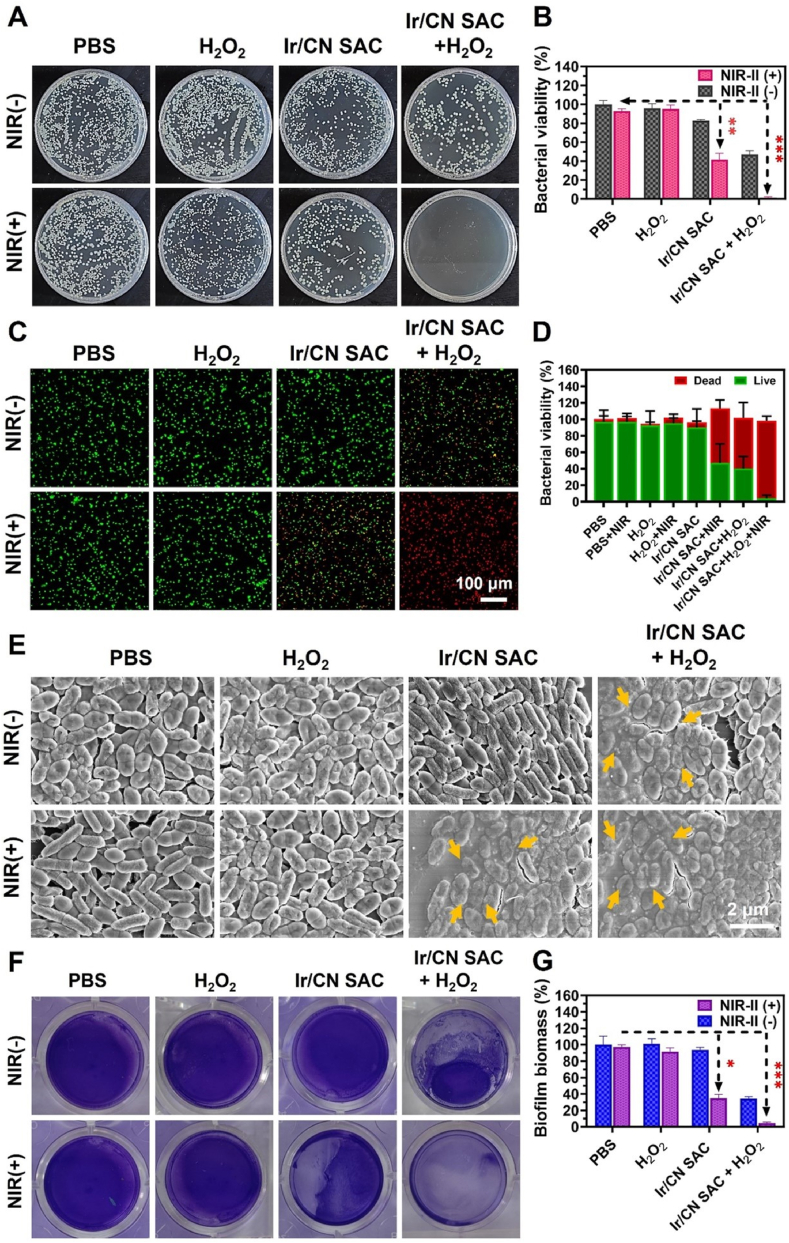
(A) Typical agar plate images of PAO1-formed bacterial colonies following different treatments. (B) Using the conventional plate counting approach, corresponding quantitative data of the PAO1 survival rate following various treatments. Green/red fluorescence indicates live/dead cells, as shown by (C) live/dead staining and (D) the corresponding relative fluorescence intensity of PAO1. (E) Normal PAO1 SEM pictures after different treatments. Bacteria with uneven morphology are indicated by yellow arrows. (F) Pictures of the PAO1 biofilm and (G) quantitative data on the biofilm's biomass after treatment. Three parallel trials served as the basis for the data (Mean ± SD, n = 3, ∗*p* < 0.05, ∗∗*p* < 0.01, ∗∗∗*p* < 0.001).

To explore the antibacterial impact, bacterial cultures were treated under different conditions and grouped into eight categories. Fig. 3, Fig. 4A showed a modest decrease in bacterial activity with Ir/CN SAC alone, likely due to the sharp lamellar structure. However, bacterial viability was notably reduced in the Ir/CN SAC + H_2_O_2_ group, reaching 33.53 % for MRSA and 47.15 % for PAO1, suggesting that Ir/CN SAC catalyzes H_2_O_2_ to generate bactericidal •OH radicals. The Ir/CN SAC + NIR-II group exhibited further bacterial reduction, with MRSA and PAO1 viability dropping to 44.09 % and 41.72 %, respectively.

In contrast, the Ir/CN SAC + H_2_O_2_ + NIR-II combination exhibited an almost complete bactericidal effect, with a 100 % and 98.6 % sterilization rate for MRSA and PAO1, respectively (Fig. 3, Fig. 4B). The results demonstrated that PTT and CDT synergistically achieved >90 % bactericidal efficacy, which was significantly higher than PTT or CDT alone. This outcome highlights the synergistic impact of NIR-II irradiation, which enhances the peroxidase-like properties of Ir/CN SAC, enabling effective chemodynamic therapy and bacterial eradication even at low concentrations. Live/dead staining (Fig. 3, Fig. 4C) and fluorescence quantification (Fig. 3, Fig. 4D) further validated this antibacterial efficacy.

SEM images revealed morphological changes in MRSA and PAO1, highlighting the bactericidal effect of Ir/CN SAC under NIR-II + H_2_O_2_. PTT is a non-invasive anti-infective method that destroys bacteria by denaturing proteins and breaking the cell membrane via NIR light-induced hyperthermia [31]. ROS produced by the nanoenzyme can disrupt biofilms and eliminate bacteria by damaging their cell walls, proteins, membranes, nucleic acids, and polysaccharides [59]. Bacteria treated with PBS and H_2_O_2_ showed little morphological change, as shown in Fig. 3, Fig. 4E. To further evaluate loss of membrane integrity, MRSA protein leakage experiments were performed using a BCA protein assay kit after various treatments (Fig. S10). Maximum protein leakage was observed in the Ir/CN SAC + H_2_O_2_ + NIR-II group, suggesting that Ir/CN SAC produces enough •OH to kill bacteria under the dual action of photothermal and peroxidase.

Both the Ir/CN SAC + NIR-II and Ir/CN SAC + H_2_O_2_ groups' initial bacterial morphology was distorted, showing clear cell shrinkage and bacterial cell membrane wrinkling. The bacterial contents were released as a result of the full disruption of the bacteria's structure and complete deformation of their original shape in the Ir/CN SAC + H_2_O_2_ + NIR-II groups. The maximum protein leakage was seen in the Ir/CN SAC + H_2_O_2_ + NIR-II group, indicating that in the presence of photothermal activity, Ir/CN SAC produces •OH to kill bacteria. These findings demonstrated that the combined action of Ir/CN SAC photothermal and peroxidase may eradicate pathogens.

Bacteria form complex extracellular polymers called biofilms, which shield the bacteria from host immune cells and drugs and help them withstand harsh conditions. In this work, MRSA biofilms following various treatments were analyzed and evaluated using the crystal violet staining technique. The more residual biofilms there were, the deeper the purple color. The Ir/CN SAC + H_2_O_2_ + NIR-II group displayed the least quantity of purple stain and the lowest OD_590_ nm value, indicating the effectiveness of this treatment in eliminating MRSA biofilms (Fig. 3F and G) and PAO1 biofilms (Fig. 4F and G). Microscopy was used to examine the MRSA cell membranes in order to learn more about Ir/CN SAC's antibacterial capabilities. PI and SYTO-9 were used in a live/dead staining assay; bacteria labeled with PI showed red fluorescence, whereas bacteria labeled with SYTO-9 showed green fluorescence. Biofilms in the Ir/CN SAC + H_2_O_2_ + NIR-II group showed strong red fluorescent patches when compared to the PBS group (Fig. 3H), indicating that this anti-biofilm technique was successful in eliminating biofilm.

### 3.5 *In vivo* evaluation of Ir/CN SAC's anti-infective efficacy

**Fig. 5:**
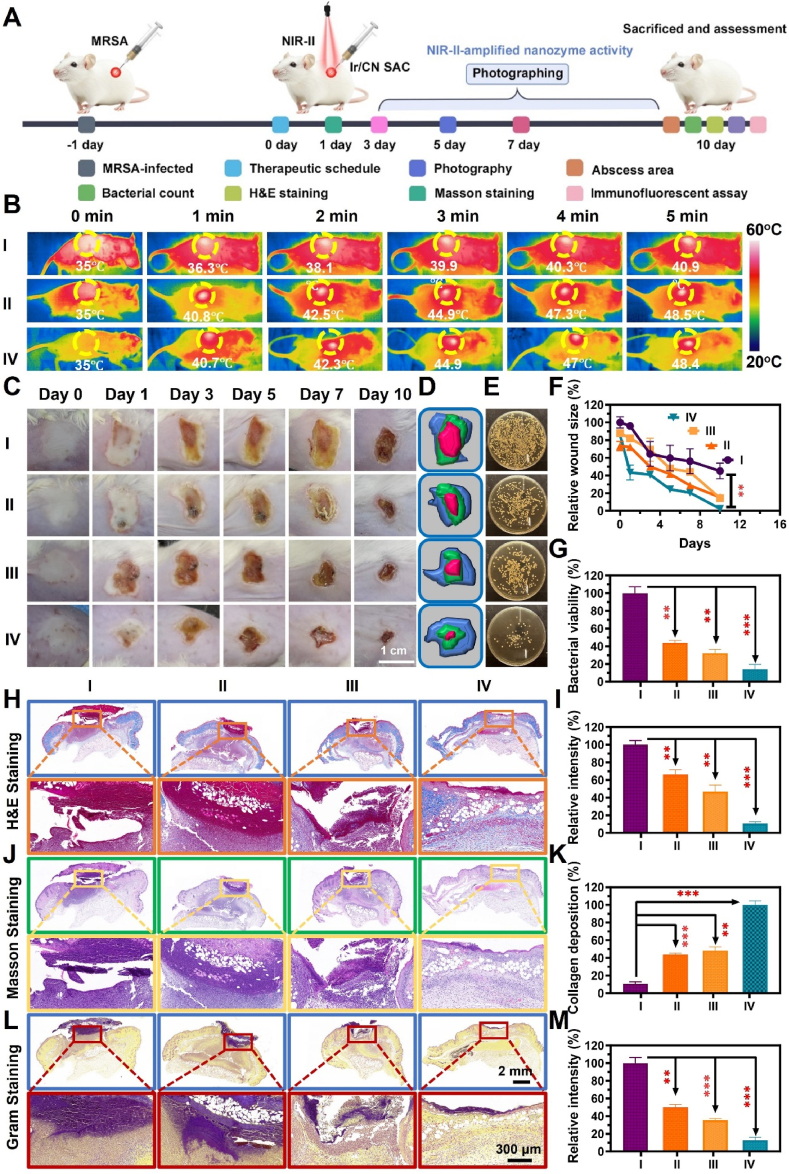
Ir/CN SAC's treatment efficacy assessment for a subcutaneous abscess infected with MRSA. (A) A schematic illustration of the treatment process. (B) Typical thermal pictures of mice exposed to laser irradiation (1270 nm, 1 W/cm^2^) for 5 min after receiving subcutaneous injections of PBS or Ir/CN SAC suspensions (100 μg/mL). (C) Pictures of abscesses infected with MRSA from different groups at different treatment intervals. I: PBS + NIR-II, II: Ir/CN SAC + NIR-II, III: Ir/CN SAC + H_2_O_2_, and IV: Ir/CN SAC + H_2_O_2_ + NIR-II. (D) A real-time representation of each therapy group's abscess healing traces. The abscess area was marked in pink, green, and blue at 0, 5, and 10 days. (E) Images of the different treatment groups' MRSA colonies. (F) The abscess size quantitative statistics over time. (G) Quantitative data pertaining to the MRSA survival rate after treatments in different groups. Following the tenth day, comprehensive histologic alterations of different therapies were stained with (H) H&E, (J) Masson, and (L) Gram. Relevant quantitative data on the percentage of (K) collagen deposition, (M) relative MRSA infection intensity, and (I) relative inflammatory intensity. Three correlative investigations (Mean ± SD, n = 3, ∗∗*p* < 0.01, ∗∗∗*p* < 0.001) served as the basis for the findings.

Building upon the antibacterial properties observed *in vitro*, we next explored the *in vivo* anti-infective potential of Ir/CN SAC. A mouse model of subcutaneous abscess was established, as shown in Fig. 5A, with temperature monitoring at the infected site using a thermal imaging device. In this experiment, Ir/CN SAC (100 μg/mL) was injected subcutaneously into the MRSA-infected region, and the area was then exposed to NIR-II (1270 nm, 1.0 W/cm^2^) for 5 min. As illustrated in Fig. 5B, the temperature at the infection site increased to 48.4 °C, indicating effective heat generation from Ir/CN SAC, which significantly exceeded the temperature of the control group, thereby confirming the compound's efficient photothermal effect in deep tissue.

We subsequently evaluated the therapeutic impact by assessing the size of the MRSA-infected tissue. Between days 0 and 3, the PBS group exhibited large abscesses, signaling active bacterial presence and heightened inflammation, which impeded healing (Fig. 5C). In contrast, the combination of Ir/CN SAC + H_2_O_2_ + NIR-II showed substantial reduction in bacterial load and enhanced wound closure, as supported by MRSA plate count data (Fig. 5E and G). This group (Ir/CN SAC + H_2_O_2_ + NIR-II) demonstrated the most significant reduction in wound size (2.36 %), compared to the other groups: PBS + NIR-II (45.0 %), Ir/CN SAC + NIR-II (14.4 %), and Ir/CN SAC + H_2_O_2_ (15.3 %) (Fig. 5D and F).

After 10 days, histological evaluation was performed on the excised tissue to investigate the cellular response. H&E staining (Fig. 5H and I) revealed heavy neutrophil infiltration in the control groups, whereas the Ir/CN SAC + H_2_O_2_ + NIR-II group showed less neutrophil activity and a greater presence of regenerating hair follicles, pointing to accelerated tissue healing. Masson's staining (Fig. 5J and K) demonstrated well-organized collagen deposition in this group, indicating extensive tissue repair. Gram staining confirmed the near-total absence of bacteria in the healed tissue (Fig. 5L and M).

**Fig. 6:**
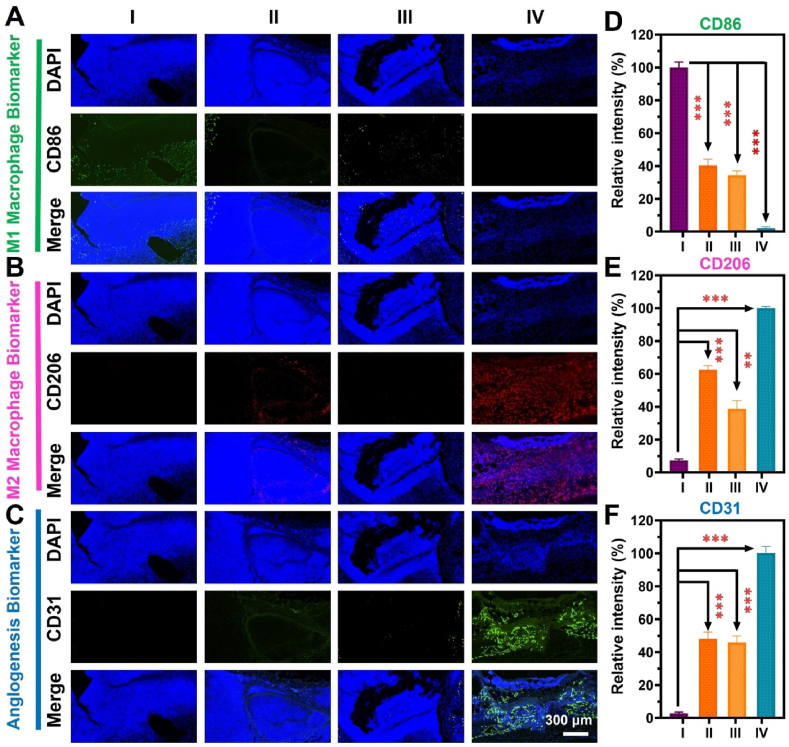
Ir/CN SAC immunofluorescence study *in vivo*. On day 10, infected tissue sections from different treatment groups were immunofluorescence stained for (A) pro-inflammatory factor (CD86), (B) anti-inflammatory factor (CD206), and (C) angiogenesis factor (CD31). I: PBS + NIR-II, II: Ir/CN SAC + NIR-II, III: Ir/CN SAC + H_2_O_2_, and IV: Ir/CN SAC + H_2_O_2_ + NIR-II. Quantitative evaluation of the different treatment groups' (D) CD86, (E) CD206, and (F) CD31 on day 10. Three correlative studies provided the results (Mean ± SD, n = 3, ∗∗*p* < 0.01, ∗∗∗*p* < 0.001).

The recovery of infected tissues is largely influenced by M1 (pro-inflammatory) and M2 (anti-inflammatory) macrophages. MRSA-infected tissue slices were immunofluorescence labeled on the tenth day to investigate the effects of Ir/CN SAC and NIR-II on M1 macrophages transitioning to the M2 phenotype. M1 and M2 macrophage markers, CD86 and CD206, were used to determine the inflammatory state in infected tissues. As seen in Fig. 6A and B, the Ir/CN SAC + H_2_O_2_ + NIR-II group had lower levels of CD86 (M1) and higher levels of CD206 (M2) compared to the other groups. M2 macrophages aid tissue regeneration by releasing angiogenic and anti-inflammatory factors. Fluorescence intensities of CD86 and CD206 for the PBS + NIR-II, Ir/CN SAC + NIR-II, Ir/CN SAC + H_2_O_2_, and Ir/CN SAC + H_2_O_2_ + NIR-II groups are shown in Fig. 6D and E. These findings suggest that Ir/CN SAC and NIR-II can promote macrophage phenotype shifts, aiding tissue repair.

Furthermore, vascularization was evaluated using CD31 staining, which showed that the Ir/CN SAC + H_2_O_2_ + NIR-II group had a significant increase in CD31-positive staining, indicating enhanced blood vessel formation (Fig. 6C). The fluorescence intensity of CD31 in the PBS + NIR-II, Ir/CN SAC + NIR-II, Ir/CN SAC + H_2_O_2_, and Ir/CN SAC + H_2_O_2_ + NIR-II groups was 2.78 %, 48.1 %, 45.9 %, and 100 %, respectively (Fig. 6F). These findings underscore the ability of Ir/CN SAC combined with NIR-II to effectively promote vascular remodeling and improve the functional state of the wound site.

### 3.6 Evaluation of Ir/CN SAC's antibacterial activity in MRSA-induced pneumonia

**Fig. 7:**
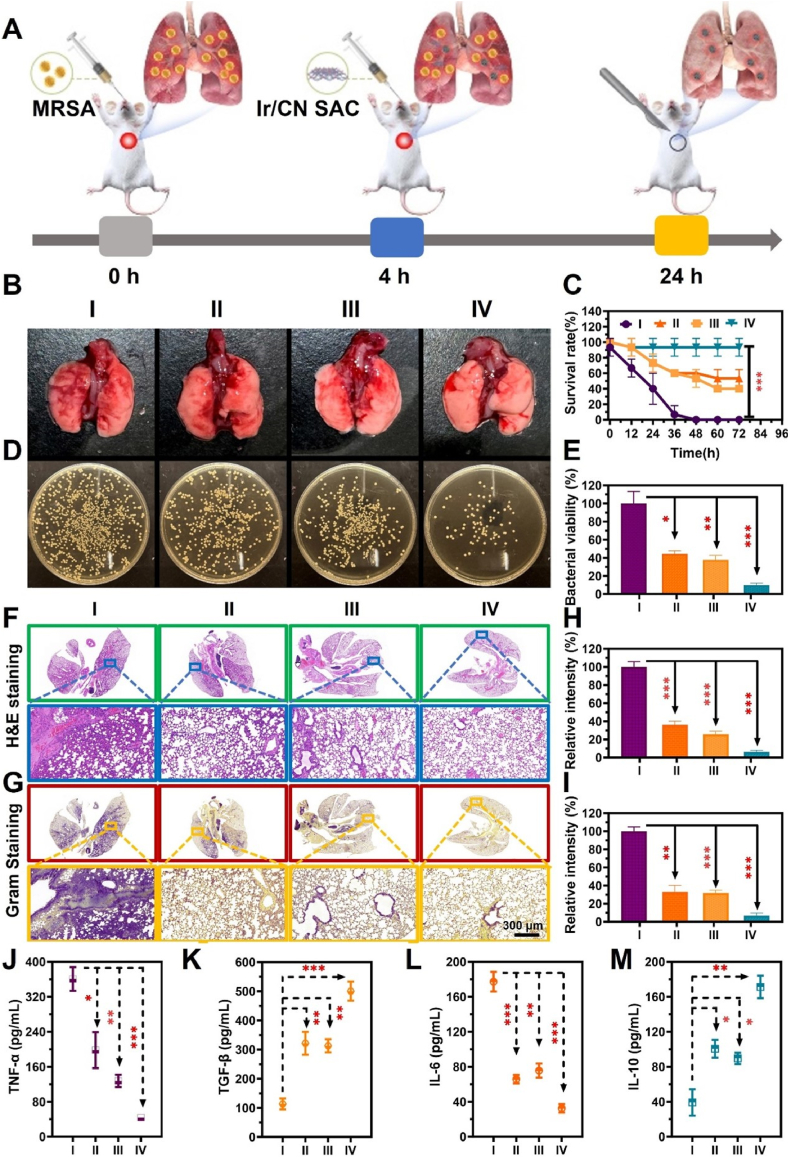
Ir/CN SAC's treatment efficacy assessment for pneumonia caused by MRSA. (A) A schematic illustration of the treatment process. (B) Pictures of lungs infected with MRSA from different groups over a 24-h period. I: PBS + NIR-II, II: Ir/CN SAC + NIR-II, III: Ir/CN SAC + H_2_O_2_, and IV: Ir/CN SAC + H_2_O_2_ + NIR-II. (C) Statistics on mice's survival rates after 72 h in various treatment groups. (D) Images of the different treatment groups' MRSA colonies. (E) Quantitative data pertaining to the MRSA survival rate after treatments in different groups. After 24 h, comprehensive histologic alterations of different therapies were shown using (F) H&E staining and (G) Gram staining. Relevant quantitative data on the relative severity of MRSA infection (I) and inflammation (H). ELISA was used to measure the amounts of (J) TNF-α, (K) TGF-β, (L) IL-6, and (M) IL-10 in lung tissue. Three correlative investigations (Mean ± SD, n = 3, ∗*p* < 0.05, ∗∗*p* < 0.01, ∗∗∗*p* < 0.001) served as the basis for the findings.

Building on the favorable *in vitro* findings regarding the biocompatibility, targeting, and antibacterial potency of Ir/CN SAC, we conducted further studies to examine its therapeutic effects in a mouse model of MRSA-induced pneumonia. As illustrated in Fig. 7A, mice were intratracheally inoculated with MRSA, and 4 h later, Ir/CN SAC (100 μg/mL) was administered (200 μL per mouse). After another 20 h, the mice were sacrificed, and their lung tissues were collected for analysis. The results in Fig. 7B show that mice treated with Ir/CN SAC + H_2_O_2_ + NIR-II for 24 h exhibited lungs that appeared pink, spongy, and with significantly fewer infection foci. In contrast, the control group (treated with PBS) showed red, mottled lungs with obvious infections. The survival rate of the treated mice was markedly higher compared to the PBS + NIR-II group (Fig. 7C).

To evaluate the bacterial load, we cultured lung tissue homogenates on TSB agar plates. The untreated group exhibited abundant bacterial growth, while the Ir/CN SAC-treated group showed a substantial reduction in bacterial colonies, confirming the potent antibacterial effect of Ir/CN SAC *in vivo* (Fig. 7D and E).

Histopathological examination of the lung tissue (Fig. 7F and H) revealed that the PBS + NIR-II group had extensive areas of pink mucus and focal infiltration by inflammatory cells. Magnified images revealed alveolar epithelial hyperplasia, thickening of the alveolar walls and septa, and alveolar collapse, all signs of significant lung damage. In contrast, treatment with Ir/CN SAC led to reduced lung consolidation, and the combination of Ir/CN SAC + H_2_O_2_ + NIR-II showed no signs of infection, suggesting near-complete bacterial eradication. Gram staining further confirmed that after 24 h of Ir/CN SAC treatment, the lungs were nearly free of bacteria (Fig. 7G and I).

We assessed the inflammatory response by measuring key cytokines such as IL-10, IL-6, TNF-α, and TGF-β, which play significant roles in the development of pneumonia. These elements transmit mechanical signals, such as tissue stiffness, affect signaling pathways through interactions between integrins and extracellular matrix proteins, and provide an immunosuppressive milieu [60]. ELISA results showed that levels of TNF-α and IL-6 were elevated in the lungs of infected mice, but these were significantly reduced in the Ir/CN SAC treatment group (Fig. 7J and L). Additionally, the levels of TGF-β and IL-10 were initially low in the infected mice, but these cytokines increased after treatment with Ir/CN SAC (Fig. 7K and M).

Finally, in Supporting Information (Figs. S11A and B), staining for CD86 (a marker of pro-inflammatory M1 macrophages) revealed reduced expression in the Ir/CN SAC + H_2_O_2_ + NIR-II group, indicating a shift toward an anti-inflammatory response. Further analysis showed a notable increase in CD206 expression (a marker for anti-inflammatory M2 macrophages) in the treated group (Figs. S11C and D). These results underscore the ability of Ir/CN SAC + H_2_O_2_ + NIR-II treatment to effectively eliminate MRSA and promote lung recovery in this model.

### 3.7 Transcriptomics Analysis of Ir/CN SAC for MRSA-induced pneumonia

**Fig. 8:**
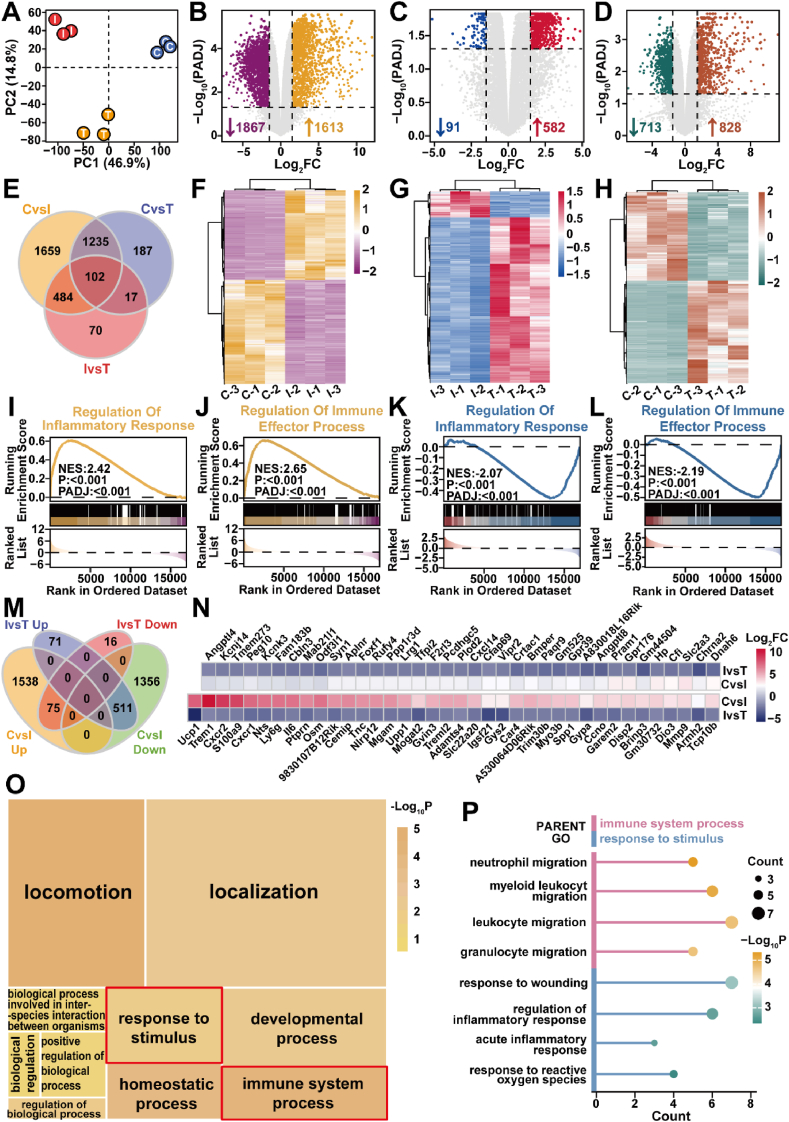
Transcriptomic analysis of lung tissues from control (C), infection (I), and treatment (T) groups. (A) Transcriptomic principal component analysis (PCA) plot with blue, red, and yellow dots representing control, infection, and treatment groups, respectively. The three volcano plots present the results of differentially expressed gene analysis between (B) I vs. C, (C) T vs. I, and (D) T vs. C groups, respectively. Grey points represent non-significantly differentially expressed genes, and points in other colors represent differentially expressed genes. (E) Venn diagram illustrating the overlap and distribution of DEGs among the I vs. C, T vs. I, and T vs. C comparisons. The three gene expression heatmaps represent the normalized expression values of 3480, 673, and 1541 differentially expressed genes in the (F) I vs. C, (G) T vs. I, and (H) T vs. C groups, respectively. (I) Visualization of the "regulation of inflammatory response" function in the results of the I vs. C GSEA enrichment analysis. (J) Visualization of the "regulation of immune effector process" function in the I vs. C GSEA enrichment analysis. (K) Visualization of the "regulation of inflammatory response" function in the T vs. I GSEA enrichment analysis. (L) Visualization of the "regulation of immune effector process" function in the T vs. I GSEA enrichment analysis. (M) Venn diagram showing the overlap of upregulated and downregulated differentially expressed genes between I vs. C and T vs. I comparisons. (N) Heatmap of Log_2_FC values for the 75 differentially expressed genes upregulated after infection and downregulated after treatment. (O) Parent GO terms treemap of the results of Gene Ontology (GO) enrichment analysis for biological processes (BP) of the above 75 genes using Metascape. The colors represent the size of the p-value, the darker the color the more significant. The size of each square represents the number of genes enriched in the corresponding function. (P) Lollipop chart illustrating the enrichment of child GO terms under the parent GO terms "response to stimulus" and "immune system process."

Lung tissue samples were divided into three groups: normal (Control, C), infection (Infection, I), and treatment (Therapy, T), and each group underwent three independent parallel experiments for transcriptomics analysis. The transcriptomics results showed that a total of 18,774 mRNAs were identified. 16,967 mRNAs were obtained for subsequent analyses after eliminating mRNAs that were barely expressed. As shown in Fig. 8A, the principal component analysis of the three sample groups illustrated the similarities and differences between them. The three groups were well separated, and the treatment group was closer to the control group on the principal component analysis (46.9 %), indicating that the treatment was effective. Subsequently, differentially expressed genes were identified using adjusted *P*_value_ < 0.05 and Log_2_FC > 1.5 as thresholds. 1613 mRNAs were upregulated and 1867 mRNAs were downregulated after infection compared to the control group (Fig. 8B and F). After treatment, 582 mRNAs were upregulated and 91 mRNAs were downregulated compared to the infected group (Fig. 8C and G). Gene set enrichment analysis (GSEA) revealed that the biological processes "regulation of inflammatory response" and "regulation of immune effector process" were significantly upregulated following infection and markedly downregulated after treatment (Fig. 8I–L). Before and after treatment, a total of 1541 mRNAs were identified as differentially expressed in the treatment group compared to the control group (Fig. 8D and H), which was much reduced compared to the post-infection changes. In addition, Appendix Fig. S12 includes a chord diagram and a heatmap that illustrate the genes mapped to these child GO terms and their expression patterns across different samples. Approximately 16.8 % (586/3480) of the mRNAs that were dysregulated after infection produced further changes in expression after treatment (Fig. 8E), of which 75 were significantly upregulated after infection and downregulated after treatment (Fig. 8M and N). GO enrichment analysis of these 75 mRNAs in biological processes (BP) using Metascape revealed clustering into 10 parent GO terms (Fig. 8O), and the child GO terms contained in the "response to stimulus" and "immune system process" categories were specifically presented (Fig. 8P), which revealed that the above mRNAs were significantly enriched in immune and inflammatory functions. These results suggest that our drug attenuates the immune and inflammatory responses of tissues during treatment.

### 3.8 Safety evaluation of Ir/CN SAC

The biosafety of Ir/CN SAC was assessed using the hemolysis assay, cell toxicity assay, and blood analysis. No notable abnormalities were found in the results of routine blood analysis (Figs. S13A-K). Additionally, there was no discernible blood toxicity as shown by the biochemical blood indexes of blood urea nitrogen (BUN), creatine kinase (CK), aspartate aminotransferase (AST), gamma-glutamyl transferase (GGT), and alanine aminotransferase (ALT) (Figs. S13L-S13P).

The cytotoxic effects of Ir/CN SAC at different concentrations (ranging from 0 to 100 μg/mL) were tested using the CCK-8 assay on L929 cells. More than 85 % of the cells remained viable, demonstrating minimal cytotoxicity and confirming the excellent biocompatibility of Ir/CN SAC (Fig. S14). Hemolysis testing at 400 mg/mL showed no evidence of hemolytic activity, which further supports the favorable blood compatibility of Ir/CN SAC (Fig. S15). Furthermore, no significant weight changes were observed in the treated mice, indicating no adverse systemic effects (Fig. S16). Examination of major organs revealed no pathological damage, suggesting that Ir/CN SAC does not cause organ toxicity (Fig. S17). These findings highlight the good biocompatibility of Ir/CN SAC, suggesting its promise for safe biomedical use.

## 4 Conclusions

In conclusion, a novel Ir/CN SAC single-atom catalyst with ultra-low metal content and fully exposed active sites was successfully synthesized. The catalyst demonstrated enhanced peroxidase-like activity (*K*_m_ = 0.69 mM) and a photothermal conversion efficiency of 50.6 %. Additionally, the outcomes demonstrated that Ir/CN SAC could successfully destroy bacteria and biofilms through photothermally enhanced biocatalysis movement. Additionally, Ir/CN SAC successfully eradicated MRSA *in vivo*, lowering the bacterial count and simultaneously accelerating the healing of acute lung damage and abscess wounds. Most significantly, the photothermally enhanced biocatalysis of Ir/CN SAC showed biocompatibility and minimal toxicity by not causing any abnormalities or impairment to normal cells and tissues. As a potential substitute for biomedical anti-infective treatments, this study concludes with an efficient combination method.

## CRediT authorship contribution statement

**Danyan Wang:** Writing – original draft, Data curation, Conceptualization. **Hui Jin:** Formal analysis, Data curation. **Yetao Shen:** Software, Methodology. **Dandan Wang:** Software. **Jingjing He:** Investigation. **Jinmiao Qu:** Project administration, Writing – review & editing. **Xiaojun He:** Writing – review & editing, Supervision, Project administration. **Zhengli Jiang:** Writing – review & editing, Supervision, Funding acquisition.

## Ethics approval and consent to participate

All animal procedures were performed in accordance with the Guidelines for Care and Use of Laboratory Animals of Wenzhou Medical University and approved by the Animal Ethics Committee of SYXK-2021–0020.

## Declaration of competing interest

The authors declare that they have no known competing financial interests or personal relationships that could have appeared to influence the work reported in this paper.
