# Supplementary material for: NIR-II-Activated iridium single-atom nanozymes for synergistic antibacterial therapy and tissue regeneration in MRSA-infected wounds and acute lung injury
Source: Bioact Mater. 2025 May 24;51:543–58. doi: 10.1016/j.bioactmat.2025.05.022 (PMC12152761; doi:10.1016/j.bioactmat.2025.05.022)
Supplement: Multimedia component 1 [file mmc1.docx]

***Supporting Information***

**Supplementary Figures**

**
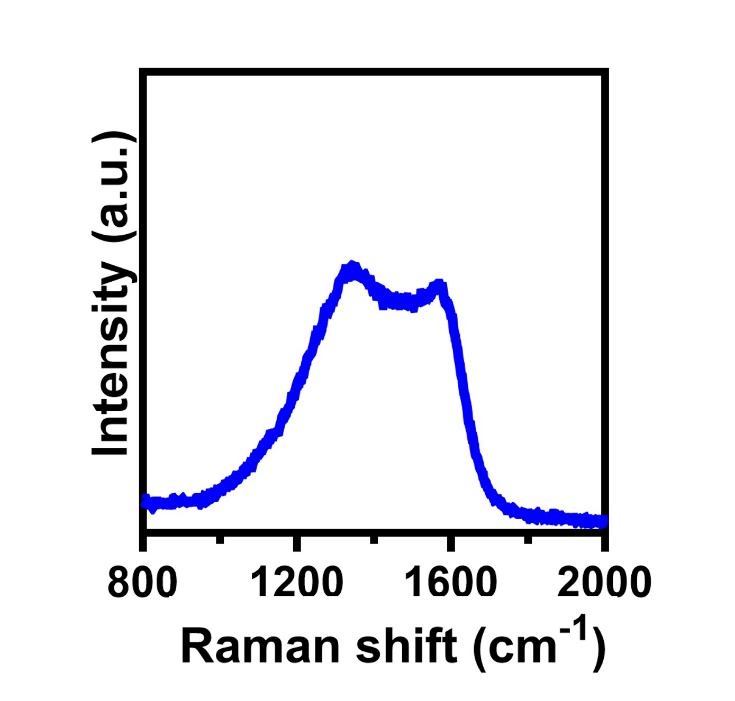
**

**Fig. S1.** Raman spectra of the Ir/CN SAC sample. Raman spectra further showed the typical D and G peaks of carbon.

**
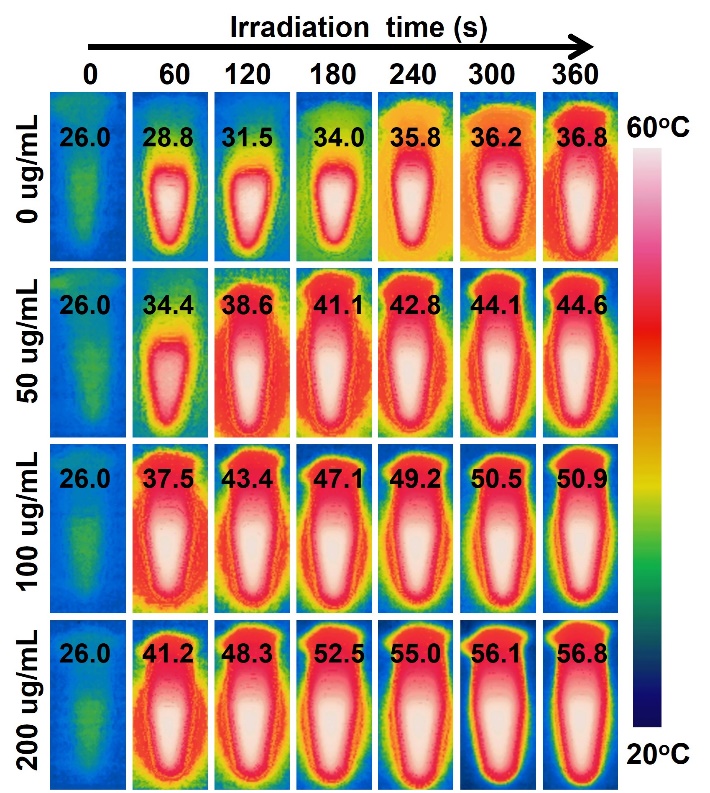
**

**Fig. S2.** Infrared thermal images of Ir/CN SAC dispersions with diverse concentrations under irradiation (1270 nm, 1 W/cm^2^).

**
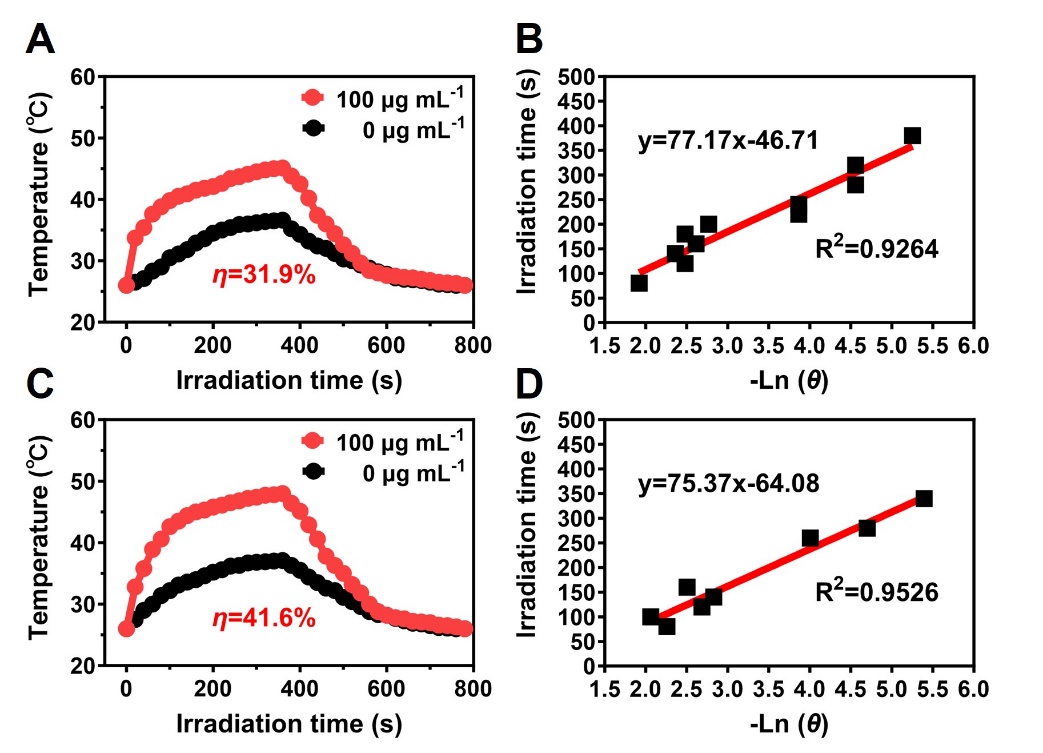
**

**Fig. S3.** Heating and cooling curves of Ir/CN SAC dispersions (0 and 100 µg/mL) at (A) 808 nm, 1 W/cm^2^ and (C) 1064 nm, 1W/cm^2^. Linear time data of (B) 808 nm, 1 W/cm^2^ and (D) 1064 nm, 1W/cm^2^ were obtained from the cooling period of Ir/CN SAC dispersions (100 µg/mL).


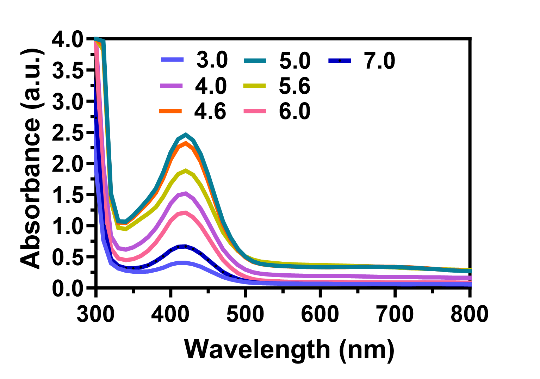


**Fig. S4.** OPD absorption at 420 nm with Ir/CN SAC dispersions (100 µg/mL) under different PH phosphate buffers.


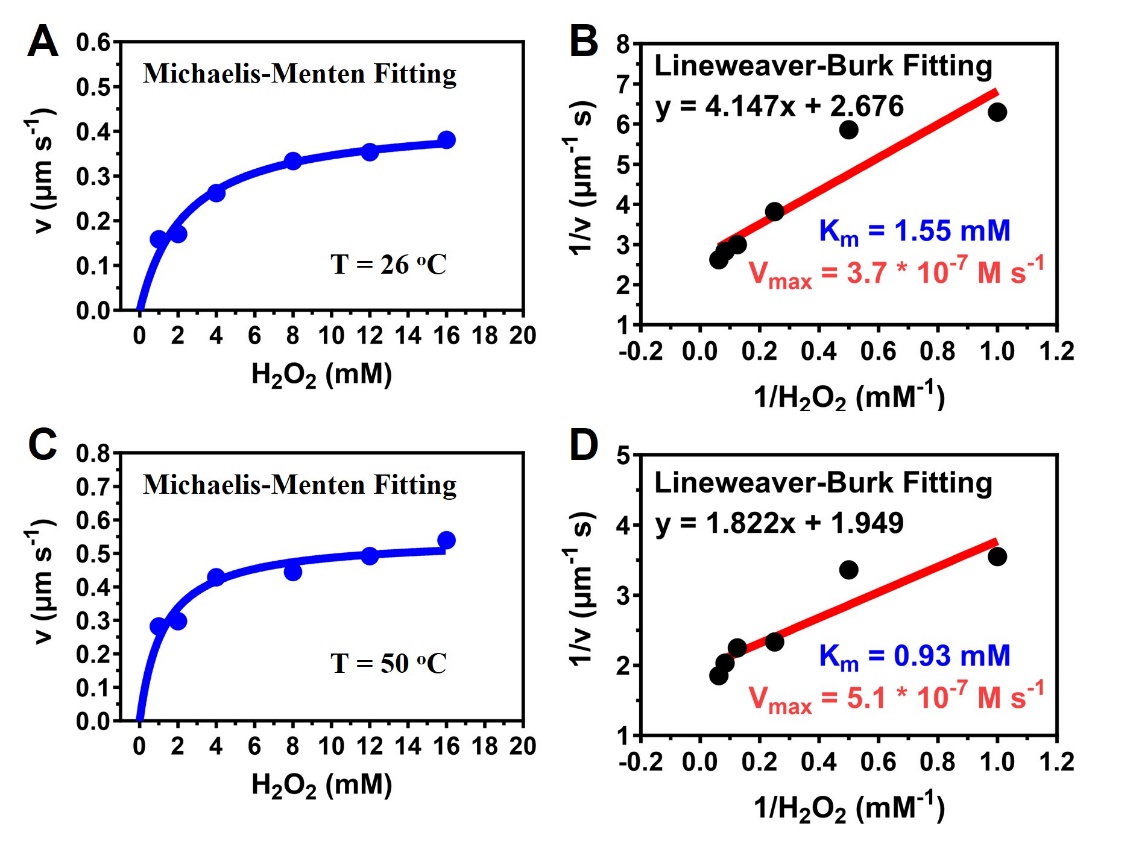


**Fig. S5.** Evaluation of kinetics. Peroxidase-like properties of Ir/CN SAC dispersions (100 µg/mL) at 26 ^o^C or 50 ^o^C with different concentrations of H_2_O_2_ (1, 6, 8, 12, 16, and 32 mM) as substrate and OPD. (A) Michaelis-Menten kinetic analysis and (B) Lineweaver–Burk plot for Ir/CN SAC with H_2_O_2_ as a substrate at 26 ^o^C. (C) Michaelis–Menten kinetic analysis and (D) Lineweaver-Burk plot for Ir/CN SAC with H_2_O_2_ as a substrate at 50 ^o^C.

**
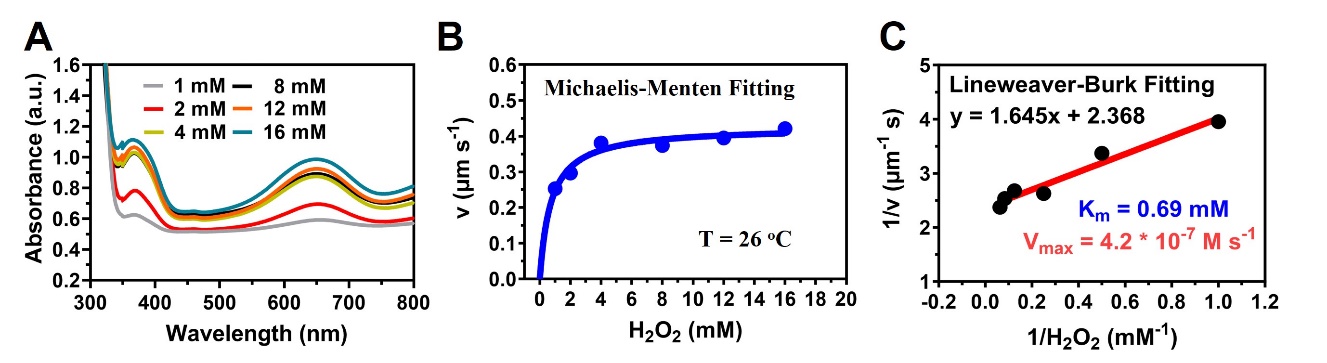
**

**Fig. S6.** Evaluation of kinetics. Peroxidase-like properties of Ir/CN SAC dispersions (100 µg/mL) at (A) 26 ^o^C with different concentrations of H_2_O_2_ (1, 2, 4, 8, 12, and 16 mM) as substrate. (B) Michaelis-Menten kinetic analysis for Ir/CN SAC with H_2_O_2_ as a substrate at 26 ^o^C. (C) Ir/CN SAC Lineweaver-Burk plot at 26 ^o^C using H_2_O_2_ as a substrate.

**Table S1.** Comparison of the kinetic parameters of Ir/CN SAC and HRP.

| **Catalysts** | **Substrates** | **K_m_ (10^-3^ M)** | **V_max_ (10^-7^ M)** | **Refs.** |
| --- | --- | --- | --- | --- |
| Horseradish peroxidase (HRP) | H_2_O_2_ | 3.70 | 0.87 | [1] |
| **Ir/CN SAC** | **H_2_O_2_** | **0.69** | **4.2** | **This work** |


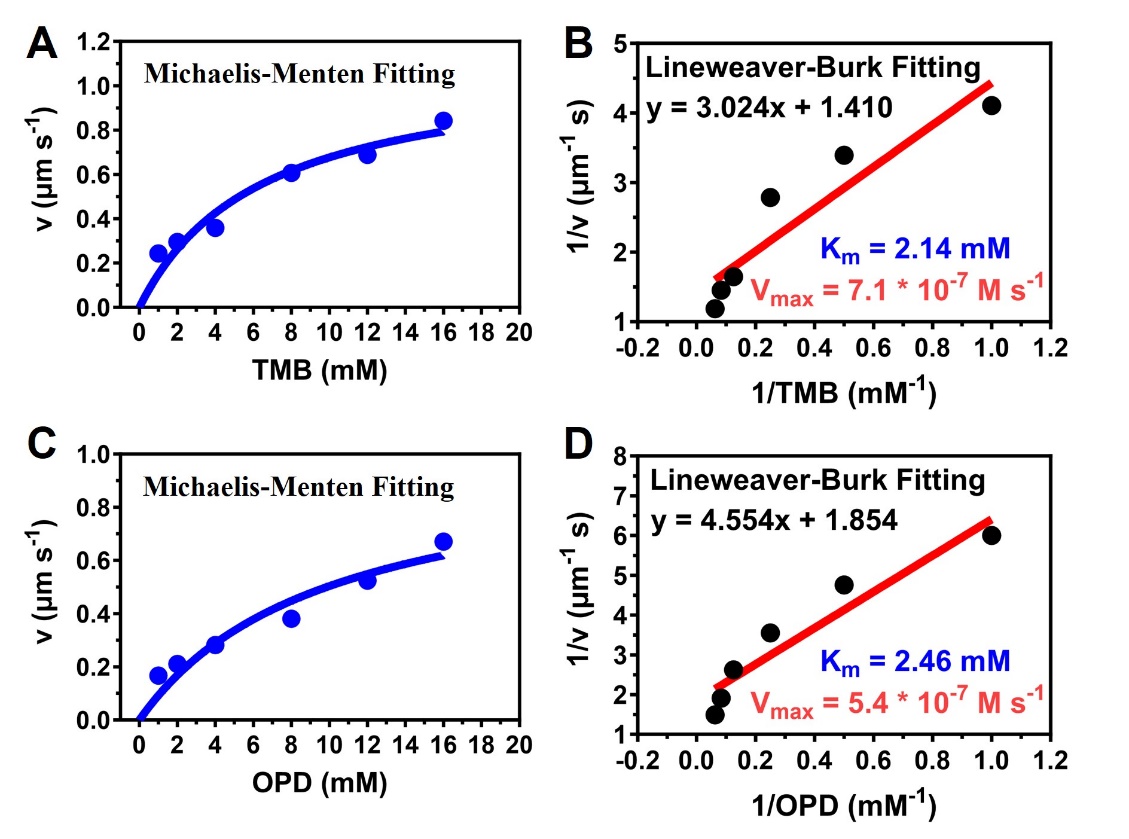


**Fig. S7.** Evaluation of kinetics. Peroxidase-like properties of Ir/CN SAC dispersions (100 µg/mL) at 26 ^o^C with different concentrations of TMB or OPD (1, 2, 4, 8, 12, and 16 mM) as substrate. (A) Michaelis-Menten kinetic analysis for Ir/CN SAC with TMB as a substrate at 26 ^o^C. (B) Ir/CN SAC Lineweaver-Burk plot at 26 ^o^C using TMB as a substrate. (A) Michaelis-Menten kinetic analysis for Ir/CN SAC with OPD as a substrate at 26 ^o^C. (B) Ir/CN SAC Lineweaver-Burk plot at 26 ^o^C using OPD as a substrate.


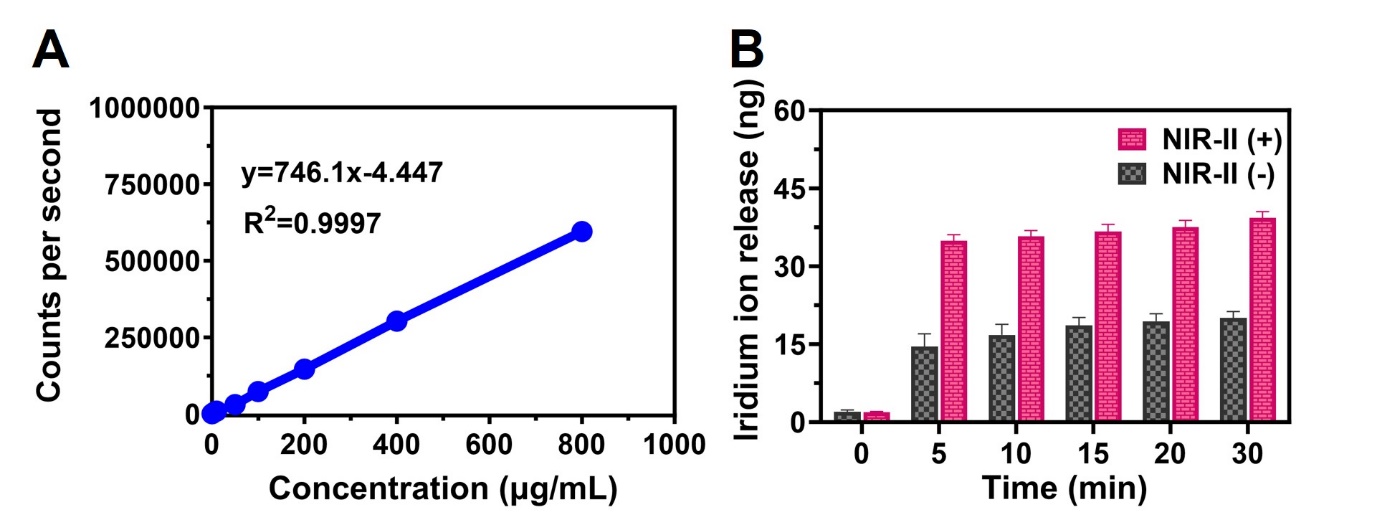


**Fig. S8.** (A) Standard curve of iridium ion release. (B) The release of iridium ions with time by ICP-MS tests before and after light exposure with 100 µL of Ir/CN SAC aqueous suspension (100 µg/mL).

**
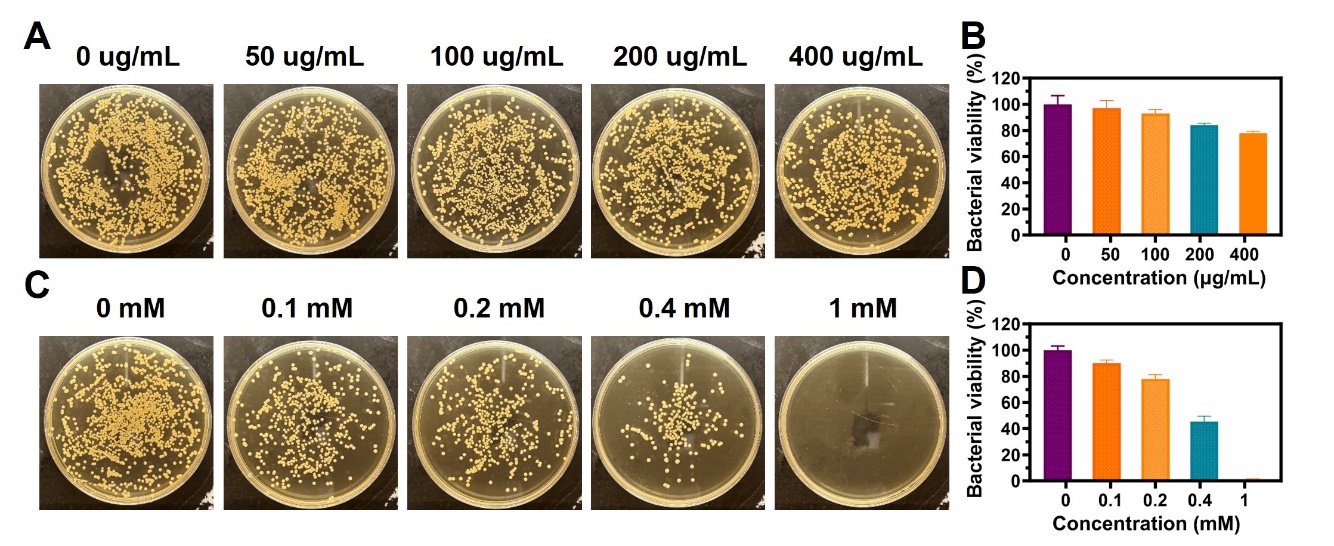
**

**Fig. S9.** MRSA inhibition effects of Ir/CN SAC dispersions (A) and quantitative data (B) at different concentrations. MRSA inhibition effects of H_2_O_2_ (C) and quantitative data (D) at different concentrations.

**
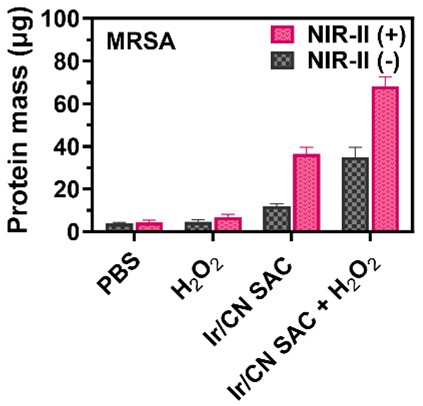
**

**Fig. S10.** The protein leakage from MRSA after different treatments using the BCA protein assay kit.

**
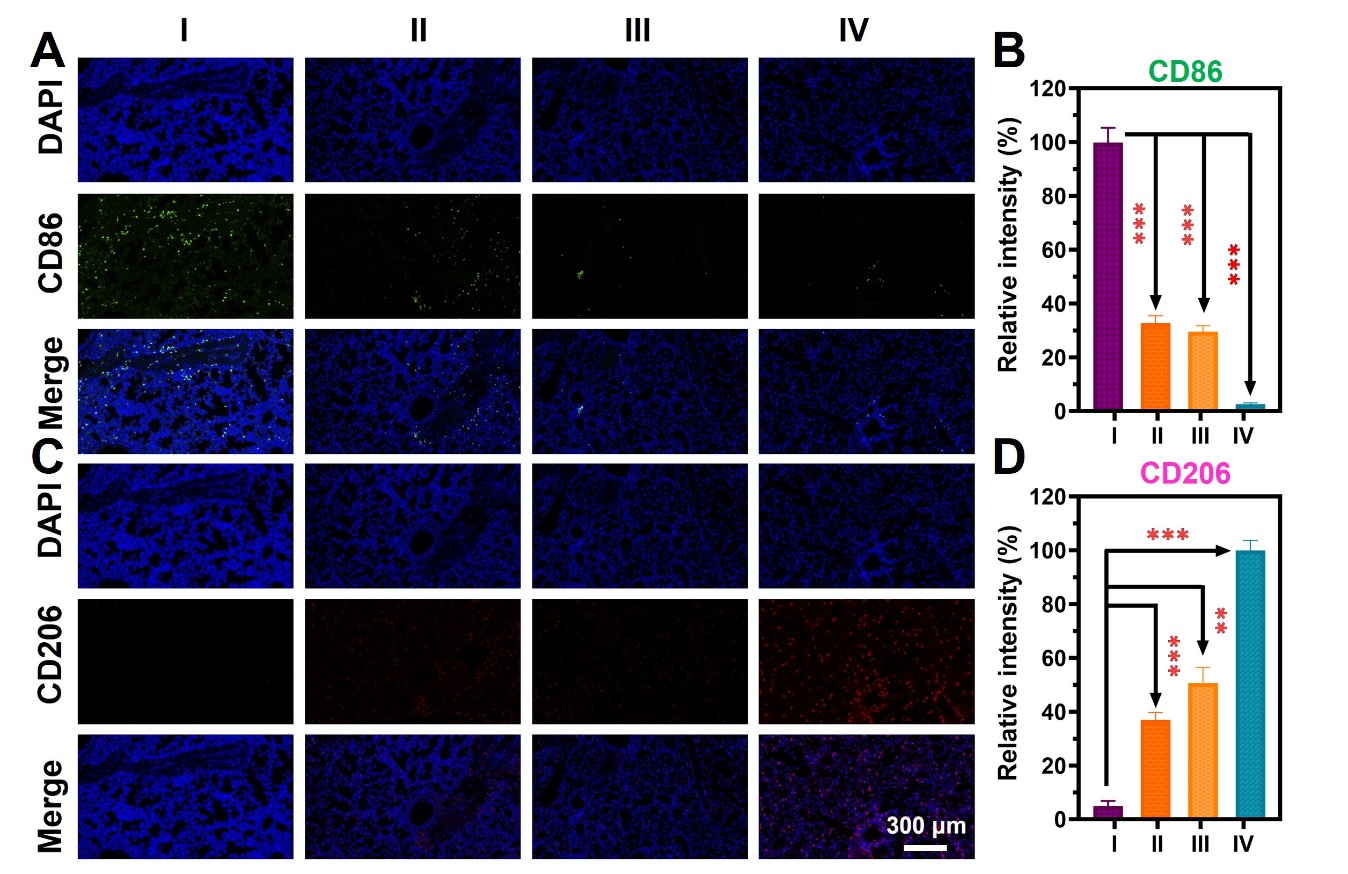
Fig. S11.** Immunofluorescence analysis of Ir/CN SAC *in vivo*. Immunofluorescence staining of infected tissue sections from various treatment groups at the 24^th^ hour for (A) CD86 (pro-inflammatory factor) and (C) CD206 (anti-inflammatory factor). I: PBS + NIR-II, II: Ir/CN SAC + NIR-II, III: Ir/CN SAC + H_2_O_2_, Ⅳ: Ir/CN SAC + H_2_O_2_ + NIR-II. Quantitative analysis of (B) CD86 and (D) CD206 of the various treatment groups at the 24^th^ hour. The data was collected from three correlative experiments (Mean ± SD, n = 3, **p < 0.01, *** p < 0.001).


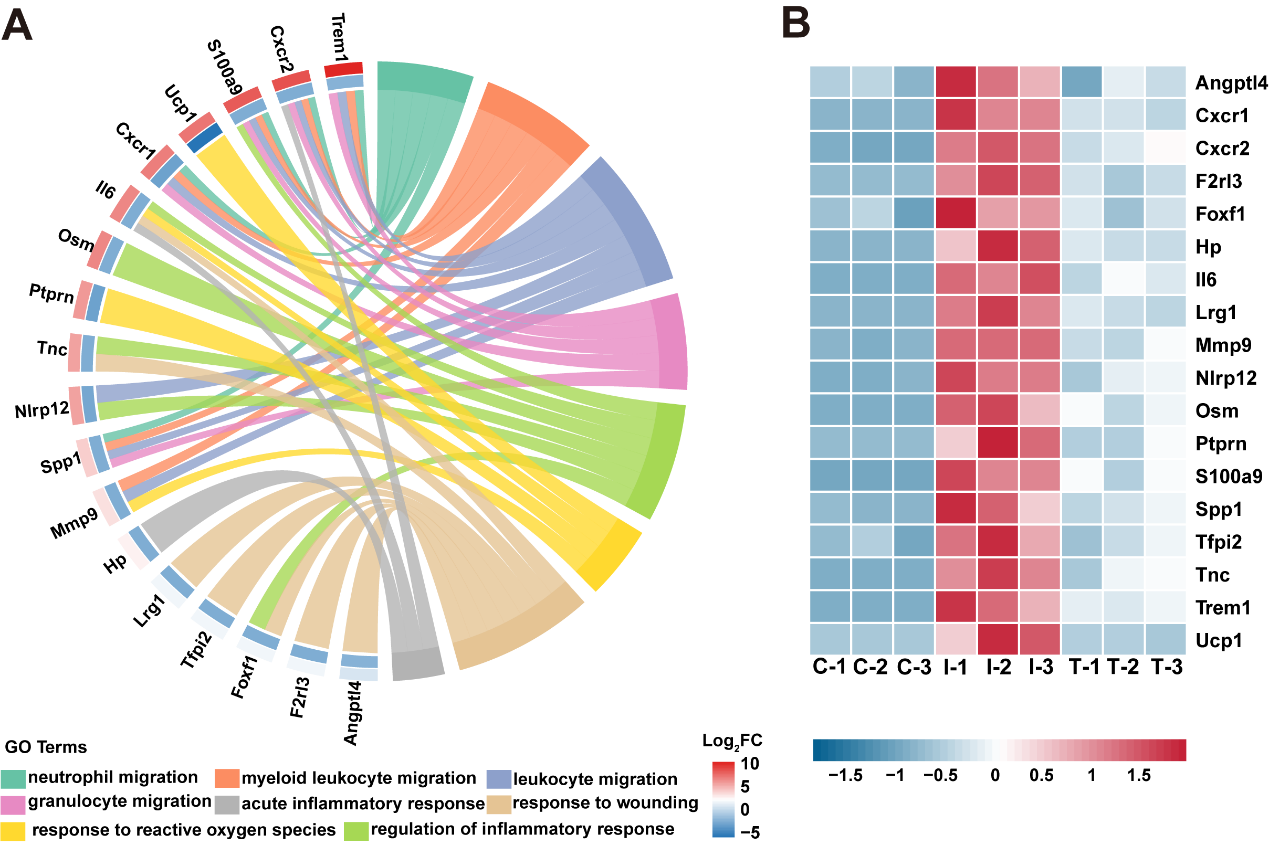


**Fig. S12.** (A) The chord diagram illustrates the mapping between significantly enriched child GO terms under the parent GO terms “response to stimulus” and “immune system process”, and their associated genes. The outer ring displays the Log_2_FC values for the comparison between I and C groups, while the inner ring represents the Log_2_FC values for the comparison between T and I groups. (B) The heatmap shows the relative expression levels of these genes across individual samples.


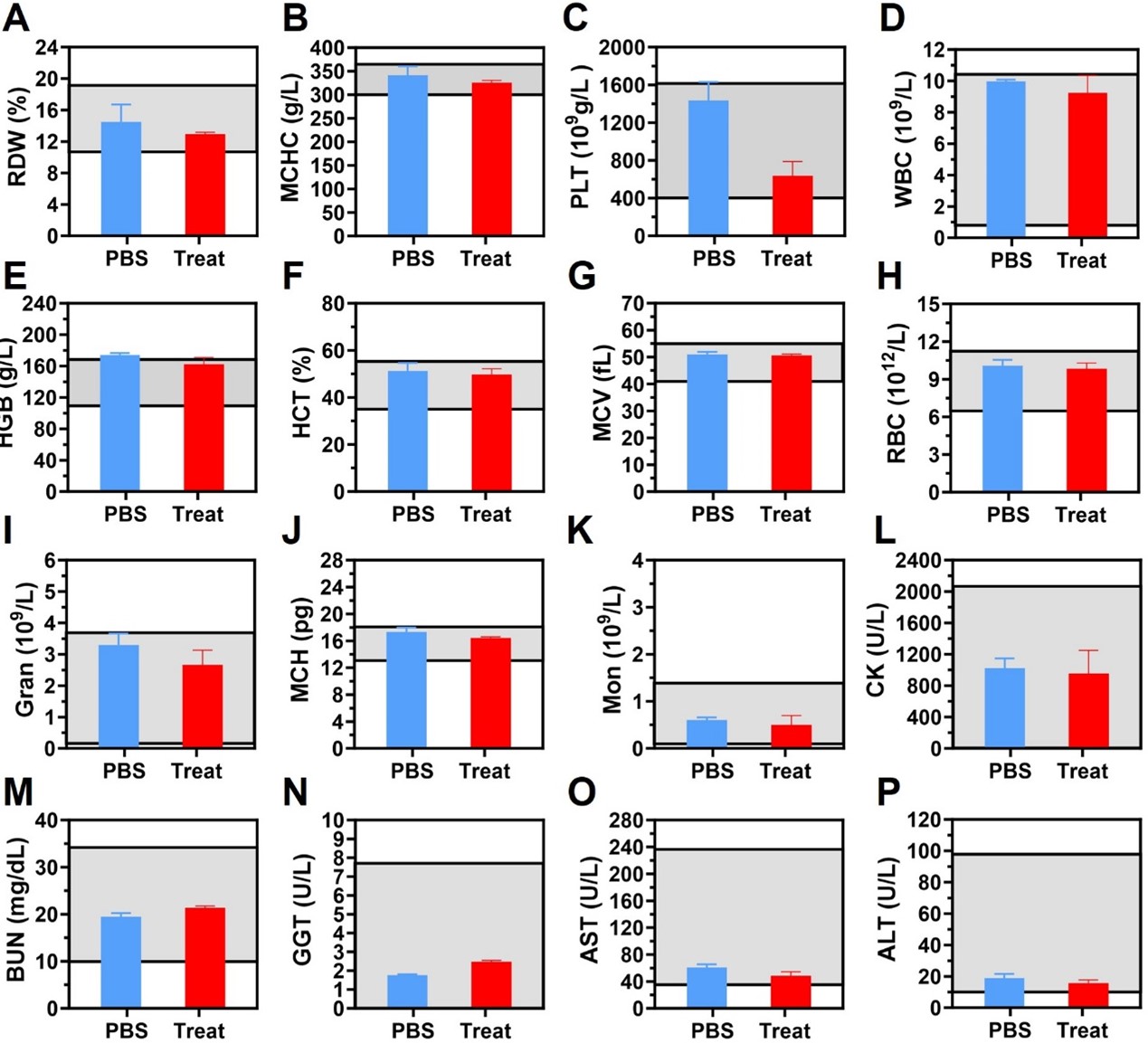


**Fig. S13.** Biosafety assessments of Ir/CN SAC. (A-K) Blood routine evaluation, (L-P) blood biochemistry evaluation of healthy mice hypodermic injection with 50 μL of Ir/CN SAC suspension (100 μg/mL) or PBS at the 24^th^ hour. The gray part is the normal range of each index of mice.


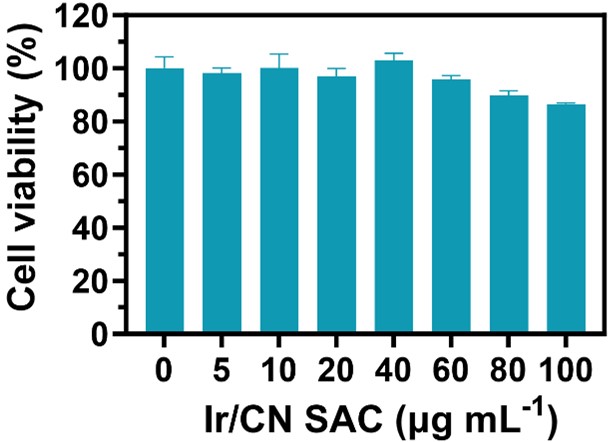


**Fig. S14.** Relative viabilities of L929 cell line after incubation with different concentrations of Ir/CN SAC (0, 5, 10, 20, 40, 60, 80, and 100 μg/mL) for 24 h.


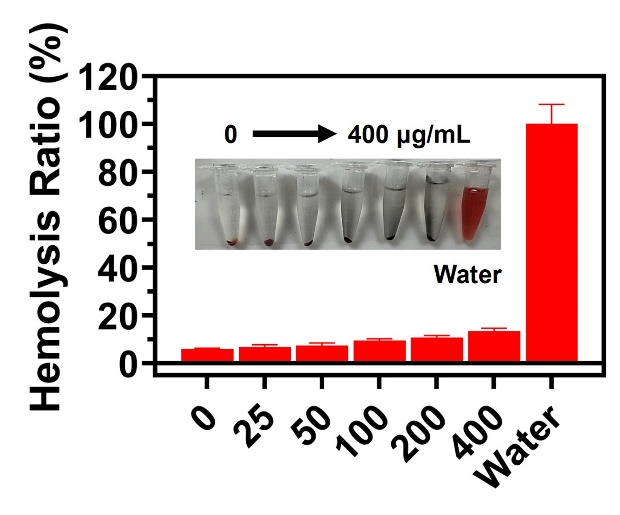


**Fig. S15.** Illustrations of hemolysis activity of Ir/CN SAC suspension of varying concentrations (0 μg/mL to 400 μg/mL) and quantitative analysis.


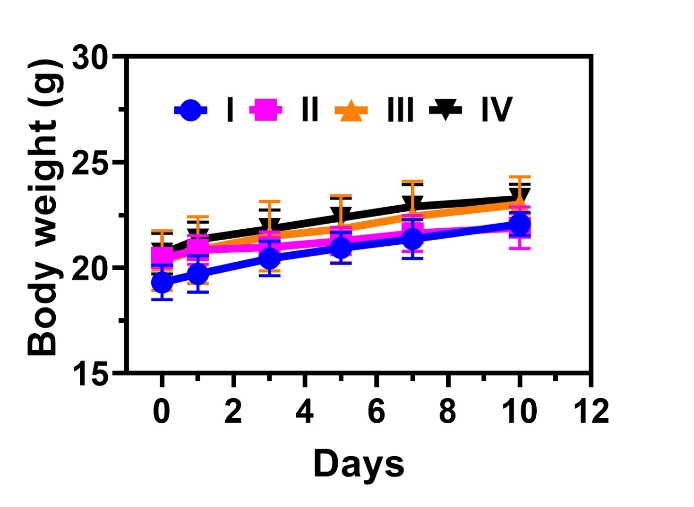


**Fig. S16.** Changes in body weight of the mice with different treatments. I: PBS + NIR-II, II: Ir/CN SAC + NIR-II, III: Ir/CN SAC + H_2_O_2_, Ⅳ: Ir/CN SAC + H_2_O_2_ + NIR-II.


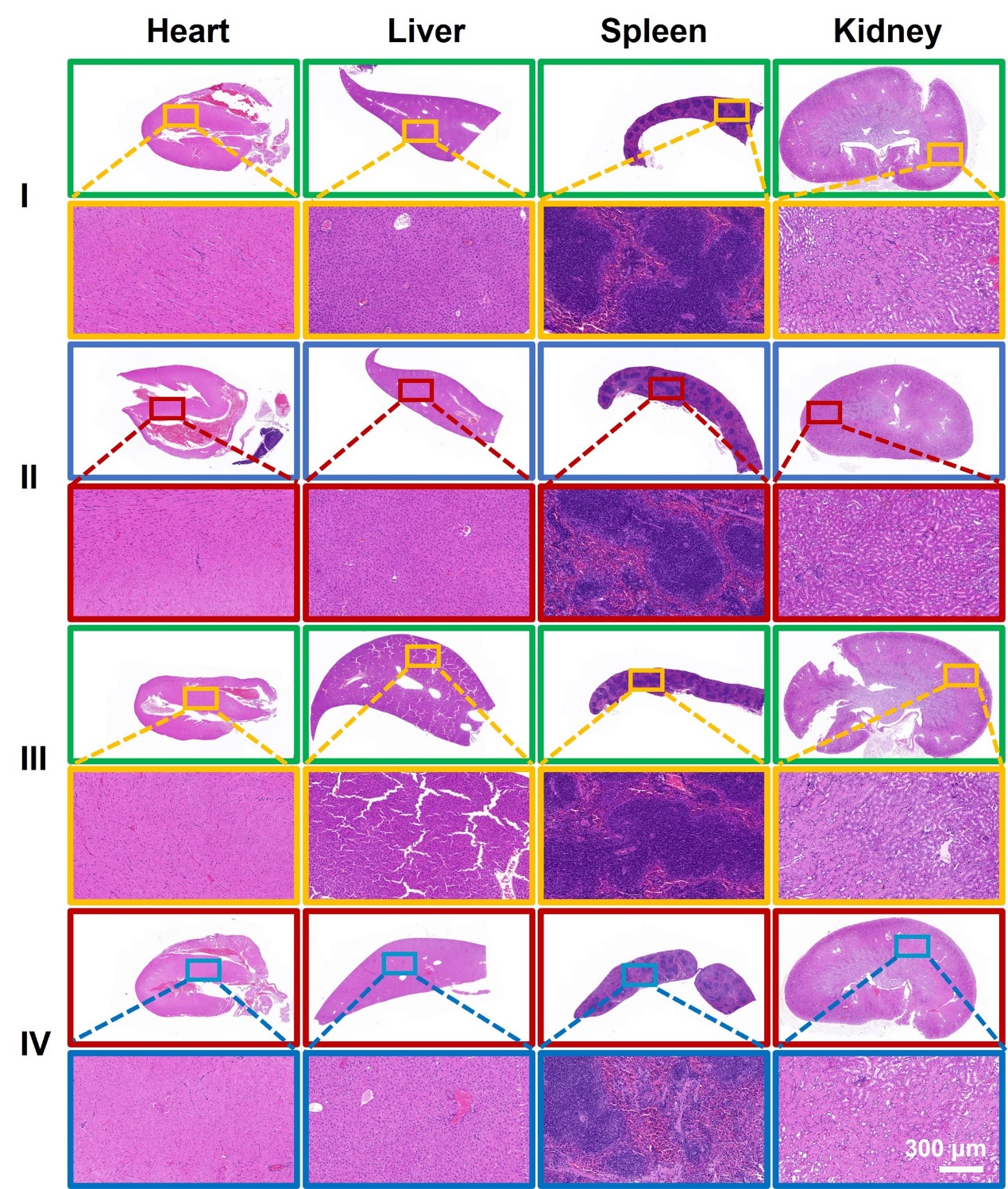


**Fig. S17.** Biosafety assay of Ir/CN SAC. H&E staining of the heart, liver, spleen, lung, and kidney at day 10 after different treatments. I: PBS + NIR-II, II: Ir/CN SAC + NIR-II, III: Ir/CN SAC + H_2_O_2_, Ⅳ: Ir/CN SAC + H_2_O_2_ + NIR-II.

**References**

[1] E.D. Brown, G.D. Wright, Antibacterial drug discovery in the resistance era, Nature 529(7586) (2016) 336-343.
